# Largest eigenvalue statistics of wavefront shaping in complex scattering media

**DOI:** 10.1038/s41467-026-77933-y

**Published:** 2026-09-30

**Authors:** Grégory Schehr, Hasan Yılmaz

**Affiliations:** 1 Sorbonne Université, Laboratoire de Physique Théorique et Hautes Energies CNRS UMR 7589, 4 Place Jussieu, 75252 Paris, Paris Cedex 05 France; 2 Department of Electrical and Computer Engineering, Saint Louis University, St. Louis, MO 63103 USA

**Keywords:** Optical physics, Statistical physics

## Abstract

In wavefront shaping, light, sound, and other waves are focused through complex scattering media onto one or more target positions, and the resulting intensity enhancement is quantified by the enhancement factor. While reproducible enhancement is crucial in experiments, the fluctuations of the enhancement factor remain largely unexplored. Here, we combine experiments, numerical simulations, and exact random-matrix theory to determine its full distribution for multi-target focusing. Exact finite-size random-matrix predictions accurately describe both the mean enhancement factor and its fluctuations beyond the asymptotic Marčenko-Pastur regime, whenever long-range mesoscopic correlations are negligible (e.g., in weakly scattering media or when only a limited number of input channels is controlled). In contrast, strongly scattering media exhibit giant enhancement-factor fluctuations that increasingly exceed these parameter-free predictions as the number of controlled input channels increases. These findings establish the enhancement factor not only as a measure of focusing performance, but also as a sensitive statistical observable that provides a simple and experimentally accessible probe of long-range mesoscopic correlations.

## Abstract (web summary)

Wavefront shaping enables waves to focus through complex scattering media. Here, authors show that focusing enhancement exhibits giant fluctuations that reveal long-range mesoscopic correlations, establishing the enhancement factor as a sensitive statistical probe of mesoscopic wave transport.

## Introduction

Wavefront shaping stands as a paradigm-shifting framework for the coherent control of light, sound, and other waves in complex scattering media, such as biological tissue and disordered materials ^1–8^. Experimental demonstrations have shown that by precisely tailoring the incident wavefront, the total transmission of microwaves, acoustic, elastic, or optical waves can be dramatically enhanced—or waves can even be tightly focused—through strongly scattering media ^1,9–19^. In optics, this level of precise coherent control over light holds profound implications for imaging ^11,20–23^, quantum secure optical authentication ^24^, high-dimensional quantum optics ^25–27^, optical manipulation ^28^, photothermal therapy ^3,29^, microsurgery ^30^, and optogenetics in complex scattering environments ^31–33^, including biological tissue, ensembles of micro- and nanoparticles, and other seemingly opaque media.

**Fig. 1:**
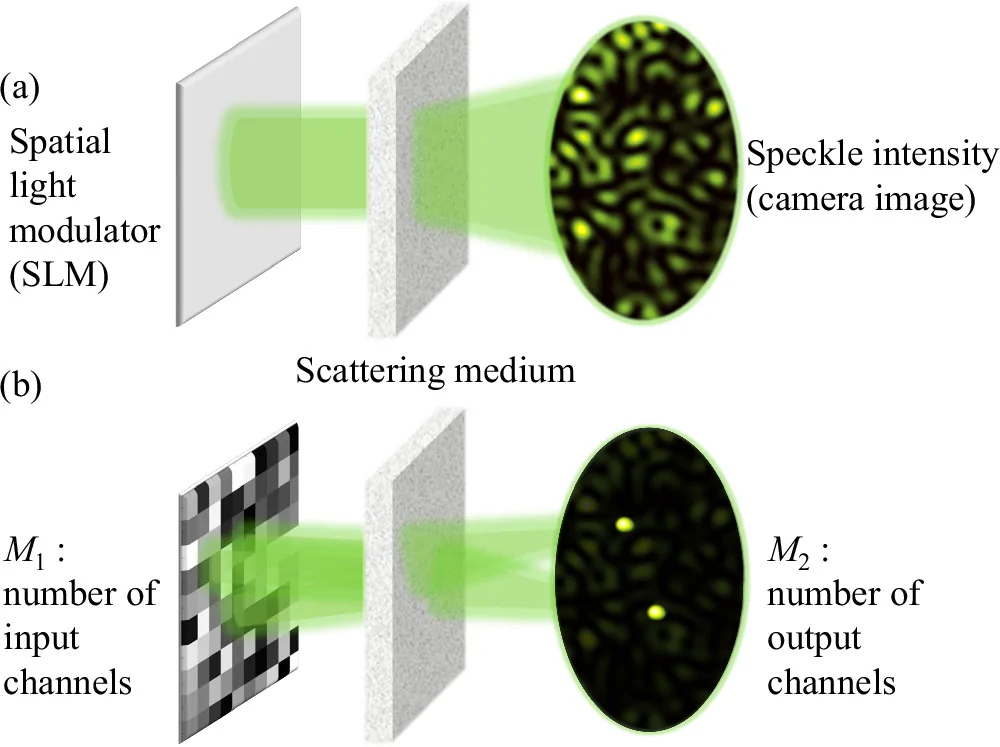
The wavefront-shaping concept to focus light through a scattering medium onto multiple target points is shown. **(a)** A sketch of the speckle pattern in transmission when an unshaped wavefront is incident onto a scattering medium is demonstrated. **(b)** The incident wavefront is shaped by a spatial light modulator with *M*_1_ degrees of freedom at the input, focusing light onto two selected target points (output channels) *M*_2_ = 2.

The measurement of the transmission matrix—encoding the input-output field relationship of a medium—is essential for controlling the output field via input wavefront shaping ^34^. Once known, the transmission matrix enables focusing light to single or multiple target positions ^1,11,17,20–28,31–42^, or transmitting images through scattering media ^43^. In particular, simultaneous focusing on multiple positions has important applications in optical metrology ^37^, optical trapping of microparticles ^28^, programmable multiport optical circuits ^25–27^, and focusing on extended guide stars ^32,33,36,44^. A key metric for evaluating the efficacy of wavefront shaping in these contexts is the enhancement factor, which quantifies the intensity enhancement achieved at the target positions ^1,4,36^. It is defined as $\eta={\sum }_{m=1}^{{M}_{2}}| \mathop{\sum }_{n=1}^{{M}_{1}}{A}_{mn}{E}_{n}^{{{\rm{opt}}}}{| }^{2}/\overline{\mathop{\sum }_{m=1}^{{M}_{2}}| \mathop{\sum }_{n=1}^{{M}_{1}}{A}_{mn}{E}_{n}}{| }^{2}$, where *A* is the *M*_2_ × *M*_1_ sub-transmission matrix of the medium, with *n* labeling the incident Fourier components at the input and *m* the position of the targets at the output. Here, *M*_1_ denotes the number of degrees of freedom at the input, while *M*_2_ denotes the number of target (focusing) points, see Fig. 1. The vector *E*^opt^ is the input wavefront that maximizes the total intensity over these *M*_2_ targets. The denominator in the definition of *η* is the same total intensity, but averaged over statistically equivalent disorder realizations (i.e., over different sub-transmission matrices *A*) or, equivalently, over independent unit-power input wavefronts *E* whose complex field components are independent and identically distributed (i.i.d.) circularly symmetric complex Gaussian entries.

One typically employs an optimization algorithm to determine the optimal input wavefront *E*^opt^ that maximizes the intensity at the target locations ^4,45^. For a single target (*M*_2_ = 1), digital optical phase conjugation attains the global maximum of the enhancement factor *η*^4^. For multiple targets (*M*_2_ > 1), the global maximum of *η* equals the largest eigenvalue ${\lambda }_{\max }$ of the focusing operator *A*^†^*A*, where *A* denotes an *M*_2_ × *M*_1_ sub-transmission matrix of the disordered medium, with *t* the normalized transmission matrix obeying Var(*t*_*mn*_) = 1/*M*_2_ (see e.g. ^17^).

In practical wavefront-shaping focusing experiments, one typically operates in the regime *M*_1_ ≫ *M*_2_. The dependence of the mean enhancement $\langle {\lambda }_{\max }\rangle$ on the number of controlled degrees of freedom *M*_1_ and output targets *M*_2_ has been observed experimentally and is widely recognized in the wavefront shaping community to exhibit the scaling $\langle {\lambda }_{\max }\rangle \sim {M}_{1}/{M}_{2}$^4,32,36,44^. On the other hand, if one assumes that the entries of *A* are independent complex Gaussian numbers, then the focusing operator is an (anti)-Laguerre-Wishart (LW) random matrix ^46^. In this case, for large *M*_1_ and *M*_2_ with fixed ratio *M*_1_/*M*_2_, the mean largest eigenvalue $\langle {\lambda }_{\max }\rangle$ coincides with the upper edge of the Marčenko–Pastur (MP) spectrum ^47–49^:

1 $$\langle {\lambda }_{\max }\rangle \,={\left(\sqrt{\frac{{M}_{1}}{{M}_{2}}}+1\right)}^{2}.$$

While the MP law predicts $\langle {\lambda }_{\max }\rangle \sim {M}_{1}/{M}_{2}$ in the limit *M*_1_ ≫ *M*_2_, the applicability of this formula (1) in experimental situations where *M*_1_ and *M*_2_ are finite remains questionable.

Furthermore, predicting the sample-to-sample and target-to-target fluctuations of the enhancement factor, i.e., $\Delta {\lambda }_{\max }\equiv \sqrt{\langle {\lambda }_{\max }^{2}\rangle -{\langle {\lambda }_{\max }\rangle }^{2}}$, is key to establishing the fundamental limits of experimental reproducibility. Yet, its full statistical distribution and fluctuations remain largely unexplored, apart from the simpler case of single-point focusing (*M*_2_ = 1), which can be analysed rather straightforwardly ^50^.

In this work, we employ a random matrix framework to describe the full distribution of the enhancement factor for focusing light onto multiple target locations (*M*_2_ > 1) through diffusive media. Under standard assumptions, the focusing operators can be described by random matrices belonging to the so-called Laguerre–Wishart ensemble ^46^. This framework accurately predicts both the mean enhancement factor and its fluctuations at finite *M*_1_ and *M*_2_, extending beyond the asymptotic Marčenko–Pastur limit and providing a parameter-free baseline for identifying excess fluctuations arising from long-range mesoscopic correlations. It is known that while long-range mesoscopic correlations do not affect the mean enhancement factor for small values of *M*_2_, they increase the background intensity, with this effect becoming experimentally observable for large values of *M*_1_^10,17,51–53^. These long-range mesoscopic correlations originate from coherent interference among multiple scattering paths and constitute one of the defining signatures of mesoscopic wave transport in disordered media ^6,46,54–62^. They manifest themselves through connected four-field correlation functions and underlie several hallmark mesoscopic phenomena, including universal conductance fluctuations ^63,64^, the emergence of open transmission channels with near-unity transmittance ^6,8,18,19,46,65,66^, and Anderson localization ^56,67,68^. They also play a fundamental role in wavefront shaping by limiting and modifying the coherent control of waves in complex scattering media ^13,15,17,18,52,65,66^. An outstanding question is whether long-range mesoscopic correlations influence the second-order statistics of the enhancement factor.

Our experiments and numerical simulations address this question by revealing correlation-induced giant fluctuations that exceed the predictions of the Laguerre–Wishart (LW) random-matrix model, thereby identifying correlation-driven deviations from the LW baseline. From a fundamental perspective, our results demonstrate that the enhancement factor is not merely a measure of focusing performance, but also a statistical observable whose fluctuations encode long-range mesoscopic correlations in wavefront-shaping experiments. This establishes enhancement-factor fluctuations as a simple and experimentally accessible probe of long-range mesoscopic correlations. From an applied perspective, the finite-size LW framework provides accurate parameter-free predictions for the enhancement factor and its fluctuations when the influence of long-range mesoscopic correlations is negligible, while providing a correlation-free baseline for identifying excess fluctuations when such correlations become significant. This framework, therefore, enables more reliable performance estimates for applications in biomedical imaging, optical metrology, optical trapping, and optical communications through scattering media.

The remainder of this paper is organized as follows. In the Results section, we first present the experimental enhancement-factor statistics and compare them with the exact finite-size Laguerre–Wishart predictions. We then introduce the random-matrix framework based on the Laguerre–Wishart ensemble and provide exact predictions for the enhancement-factor statistics. Next, we investigate the role of long-range mesoscopic correlations by combining numerical simulations, analytical predictions, and experimental measurements, demonstrating that these correlations give rise to giant fluctuations in the enhancement factor beyond the Laguerre–Wishart baseline. In the Discussion section, we place our findings in a broader context and summarize the main results. Finally, the “Methods” section provides details of the experimental transmission-matrix measurements and wave-propagation simulations.

## Results

### Transmission-matrix measurements and enhancement-factor statistics

The transmission matrix of a scattering medium provides the information required to modulate the incident wavefront with a spatial light modulator (SLM) and focus light onto single or multiple target points, as illustrated in Fig. 1: an unmodulated beam produces a random speckle pattern [Fig. 1a], whereas a shaped input beam enables simultaneous focusing at multiple target points [Fig. 1b].

Experimentally, we measure the transmission matrix *t* of a scattering sample using phase-shifting interferometry ^69^, see Supplementary Note 3 for a sketch of the experimental setup. The scattering sample consists of a densely packed zinc oxide (ZnO) nanoparticle layer on a cover slip, with a thickness of approximately 10 μm, much larger than the transport mean free path *l*_*t*_ = 1μm, ensuring that light transport within the ZnO layer is diffusive. In our measured *t*_*mn*_, the input channel *n* corresponds to a 9 × 9 SLM segment (≈one Fourier component on the front surface). The output channel *m* corresponds to a position on the back surface, i.e., *t*_*mn*_ maps the SLM field to the outgoing field at the back surface.

Starting from the experimental transmission matrix *t*_*mn*_, we generate an ensemble of focusing operators *A*^†^*A*, with *A* denoting a randomly chosen sub-transmission matrix of size *M*_2_ × *M*_1_. We work in the regime *M*_1_ ≫ *M*_2_, with ${M}_{1}\in [100,1000]\cap {\mathbb{Z}}$ and ${M}_{2}\in [1,5]\cap {\mathbb{Z}}$. Each sub-matrix *A* is formed by randomly selecting *M*_1_ input segments on the SLM and *M*_2_ output target focusing positions *m* in transmission. For each sub-matrix size *M*_2_ × *M*_1_, we compute its eigenvalues via *A*^†^*AV*_*α*_ = *λ*_*α*_*V*_*α*_. We then determine the probability density ${P}_{\max }({\lambda }_{\max })$, the mean $\langle {\lambda }_{\max }\rangle$, and the standard deviation $\Delta {\lambda }_{\max }$ of the largest eigenvalue of the associated focusing operator, thereby obtaining the experimental statistical distribution of the enhancement factor for light focusing through the scattering sample.

**Fig. 2:**
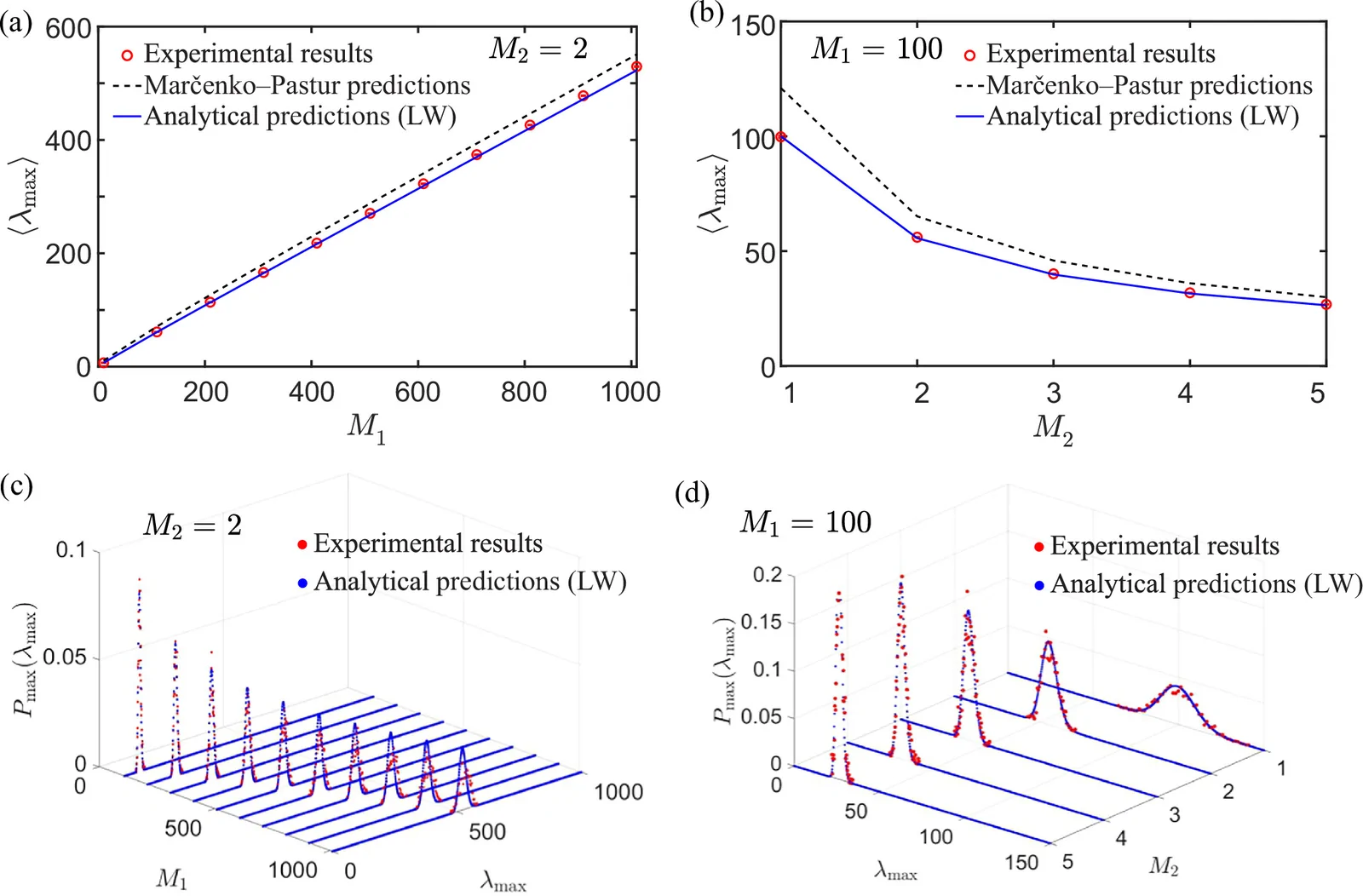
The mean and probability density of the enhancement factor ${\lambda }_{\max }$ are shown. The mean enhancement factor $\langle {\lambda }_{\max }\rangle$ is plotted versus the number of input channels *M*_1_ at fixed *M*_2_ = 2 (**a**) and versus the number of output channels *M*_2_ at fixed *M*_1_ = 100 (**b**). The predictions of the finite-size Laguerre--Wishart (LW) ensemble (solid blue), obtained from the exact analytical expression, are in quantitative agreement with the experiments (red open circles). In contrast, the Marčenko--Pastur prediction (dashed black) lies well outside the experimental uncertainty in panels (**a**, **b**). The error bars, computed from three independent experimental transmission-matrix measurements, are smaller than the open-circle symbols. With no fitting parameter, the predicted probability densities $P({\lambda }_{\max })=Q{{\prime} }_{\max }({\lambda }_{\max })$ from Eq. (4) likewise match quite well the experimental distributions for small *M*_1_ in (**c**) and for all values of *M*_2_ for *M*_1_ = 100 in (**d**).

In Fig. 2, we present the experimental and theoretical mean largest eigenvalues $\langle {\lambda }_{\max }\rangle$ (i.e., the enhancement factor *η*) and their probability densities ${P}_{\max }({\lambda }_{\max })$ for various numbers of input (*M*_1_) and output (*M*_2_) channels. The scaling of $\langle \lambda_{\max} \rangle$ with *M*_1_ [Fig. 2a] and with *M*_2_ [Fig. 2b] predicted by the LW random-matrix-based analytical calculation is in excellent agreement with the experimental values, whereas the Marčenko–Pastur prediction (1) shows a clear deviation. This deviation arises because the Marčenko–Pastur law is exact only in the asymptotic limit of large *M*_1_ and *M*_2_, while the LW random-matrix-based analytical calculation remains accurate for finite *M*_1_ and *M*_2_. Moreover, the LW-based random matrix theory predicts not only the mean of $\lambda_{\max}$ but also its full distribution ${P}_{\max }({\lambda }_{\max })$, as shown in Fig. 2c, d. The theoretical prediction of ${P}_{\max }({\lambda }_{\max })$ agrees well with the experimental distribution at small *M*_1_. As *M*_1_ increases, however, the experimentally observed distribution becomes progressively broader than the Laguerre–Wishart prediction (Fig. 2c). Within the correlation-free Laguerre–Wishart framework, this systematic excess broadening is the first indication that long-range mesoscopic correlations contribute to enhancement-factor fluctuations. This interpretation is consistent with the well-established increase of long-range mesoscopic correlations with the increasing number of controlled input channels *M*_1_^10,15,17,52,70^. For *M*_1_ = 100, the experimental and theoretical probability densities remain in close agreement for all investigated values of ${M}_{2}\in [1,5]\cap {\mathbb{Z}}$ (Fig. 2d).

### Random-matrix framework: the Laguerre-Wishart ensemble

Having presented the experimental results for the mean enhancement factor and its probability distributions, together with their comparison to the analytical predictions, we now detail the random-matrix framework from which these predictions are derived. Our approach is based on an analytic treatment of the spectral properties of the focusing operator within a random-matrix framework. To this end, we model the entries of the *M*_2_ × *M*_1_ sub-transmission matrix *A* as independent complex Gaussian variables. This is the standard statistical description of open scattering systems that support a large number of channels, in which the coherent superposition of many scattering paths gives rise to fully developed speckle and approximately Gaussian field statistics ^71–74^. The resulting Gaussian model provides the simplest parameter-free description of enhancement-factor statistics in the absence of long-range mesoscopic correlations, serving as a quantitative reference for identifying correlation-induced excess fluctuations. Under this assumption, the focusing operator *A*^†^*A* belongs to the complex anti–LW ensemble ^46^, where “anti” indicates that the underlying matrix *A* is rectangular with more columns than rows (*M*_1_ > *M*_2_) ^75–77^. Consequently, *A*^†^*A* is an *M*_1_ × *M*_1_ random matrix whose spectrum consists of exactly *M*_2_ positive eigenvalues ${\lambda }_{1},\ldots,{\lambda }_{{M}_{2}}$, while the remaining *M*_1_ − *M*_2_ eigenvalues are identically zero.

These nonzero eigenvalues encode the strength of the transmission channels. Since the matrix *A* is random, so are these nonzero eigenvalues, which thus fluctuate from one sample of *A* to another. However, it turns out that their joint (i.e., multivariate) statistics are known exactly. Indeed, their joint probability density function (JPDF) reads ^75,76^

2 $$P({\lambda }_{1},\ldots,{\lambda }_{{M}_{2}})=\frac{1}{Z}\mathop{\prod }_{i=1}^{{M}_{2}}{\lambda }_{i}^{\nu }\,{e}^{-{M}_{2}{\lambda }_{i}}{\prod}_{i < j}{({\lambda }_{i}-{\lambda }_{j})}^{2},$$

where *ν* = *M*_1_ − *M*_2_ > 0 and *Z* is a normalization factor that can be computed explicitly. From this JPDF (2), the average density of eigenvalues, defined as $\rho (\lambda )={\sum }_{i=1}^{{M}_{1}}\langle \delta (\lambda -{\lambda }_{i})\rangle$ where *δ* is a Dirac delta-function and 〈 ⋯ 〉 denotes an average over the JPDF in (2), can be computed explicitly. It is simply obtained by integrating the JPDF (2) over ${\lambda }_{2},\cdots \,,{\lambda }_{{M}_{2}}$ while keeping *λ*_1_ ≡ *λ* fixed. This multiple integral can be carried out explicitly and it reads $\rho (\lambda )=\nu \delta (\lambda )+\tilde{\rho }(\lambda )$ where the density of nonzero eigenvalues is given by (see e.g. ^78^)

3 $$\tilde{\rho }(\lambda )={M}_{2}^{\nu+1}{\lambda }^{\nu }{e}^{-{M}_{2}\lambda }\mathop{\sum }_{k=0}^{{M}_{2}-1}\frac{k!}{(k+\nu )!}{[{L}_{k}^{(\nu )}({M}_{2}\lambda )]}^{2}$$

where ${L}_{k}^{(\nu )}(\lambda )$ is the generalized Laguerre polynomial of index *ν* and degree *k*.

Of particular interest is the statistics of the largest eigenvalue ${\lambda }_{\max }=\max \{{\lambda }_{1},\ldots,{\lambda }_{{M}_{2}}\}$, which in the context of wavefront shaping corresponds directly to the enhancement factor ^17^. Rather than computing its probability density function ${P}_{\max }(x)$ directly, it is often more convenient to consider the cumulative distribution function ${Q}_{\max }(x)={{\rm{Prob}}}({\lambda }_{\max }\le x)=\int_{0}^{x}{P}_{\max }(x^{\prime} )\,dx^{\prime}$^79^. Indeed, the event ${\lambda }_{\max }\le x$ is equivalent to all eigenvalues satisfying *λ*_*i*_≤*x* for *i* = 1, …, *M*_2_. Therefore, ${Q}_{\max }(x)$ can be obtained by integrating the joint probability density function in (2) over the hypercube ${[0,x]}^{{M}_{2}}$. This multiple integral can be evaluated explicitly using standard techniques from random matrix theory and determinantal processes, yielding (see also ^80,81^)

4 $${Q}_{\max }(x)={A}_{{M}_{1},{M}_{2}}{{\det }_{1\le i,j\le {M}_{2}}}\left[\gamma (i+j-1+\nu,{M}_{2}x)\right]\,$$

where $\gamma (s,z)=\int_{0}^{z}{t}^{s-1}{e}^{-t}\,dt$ is the lower incomplete gamma function (which in our case can be simply expressed in terms of exponentials and polynomials) and ${A}_{{M}_{1},{M}_{2}}={M}_{2}!/(\mathop{\prod }_{k=0}^{{M}_{2}-1}(k+1)!\,(k+\nu )!)$ is a normalization constant (independent of *x*). As *x* → 0 it behaves as ${Q}_{\max }(x) \sim {x}^{{M}_{1}{M}_{2}}$ while $1-{Q}_{\max }(x) \sim {x}^{{M}_{1}+{M}_{2}-2}\,{e}^{-{M}_{2}x}$ as *x* → *∞*.

This *M*_2_ × *M*_2_ determinant in Eq. (4) is straightforward to evaluate numerically (for moderate values of *M*_2_), allowing a direct computation of the mean $\langle {\lambda }_{\max }\rangle$ and fluctuations $\Delta {\lambda }_{\max }$. This exact characterization (4) is particularly useful in wavefront shaping experiments, where *M*_2_ is typically small. As discussed in the previous subsection, Fig. 2 revisits the behavior of the largest eigenvalue, but now emphasizes the exact finite-size analytical predictions. In Fig. 2a, b, we use Eq. (4) to compute the theoretical curves for $\langle {\lambda }_{\max }\rangle$ and compare them with the experimental values, finding excellent agreement for all investigated *M*_1_ and *M*_2_. At the same time, these panels clearly reveal a deviation from the MP prediction (1), which is expected since the MP law strictly applies in the limit where both *M*_1_ and *M*_2_ are large, whereas here 2 ≤ *M*_2_ ≤ 5. In Fig. 2c, d, we further use ${P}_{\max }(x)=d{Q}_{\max }(x)/dx$ with ${Q}_{\max }(x)$ given in Eq. (4) to generate the analytical curves for the full distribution of ${\lambda }_{\max }$ and compare them with the experimentally obtained histograms, finding excellent agreement for small *M*_1_.

In wavefront shaping experiments, one is often interested in the limit where *M*_1_ ≫ *M*_2_. In this limit, one can show that the complex LW ensemble in (2) converges, up to a rescaling of the eigenvalues by *M*_1_ after centering around *M*_1_/*M*_2_ and rescaling by ${M}_{2}/\sqrt{2{M}_{1}}$, to the so-called Gaussian Unitary Ensemble (GUE) that describes Hermitian matrices with independent (complex) Gaussian entries. Consequently for large *M*_1_ ≫ *M*_2_ one finds

5 $$\langle {\lambda }_{\max }\rangle \approx \frac{1}{{M}_{2}}\left({M}_{1}+\sqrt{2\,{M}_{1}}\,\langle {\mu }_{\max }\rangle \right)\,$$

where $\langle {\mu }_{\max }\rangle$, which only depends on *M*_2_ and not on *M*_1_, denotes the average value of the largest eigenvalue of a GUE matrix of size *M*_2_ × *M*_2_. For example, one finds $\langle {\mu }_{\max }\rangle=\{0,\sqrt{2/\pi },27/(8\sqrt{2\pi })\}$ for *M*_2_ = {1, 2, 3} respectively (see Supplementary Note 1 for further details). In the limit *M*_2_ ≫ 1, one has $\langle {\mu }_{\max }\rangle \approx \sqrt{2{M}_{2}}$ and this formula (5) coincides at leading order with the Marčenko-Pastur prediction given in Eq. (1) in the main text, in the limit *M*_1_/*M*_2_ ≫ 1. However, for finite *M*_2_, the average value $\langle {\mu }_{\max }\rangle$ differs from $\sqrt{2{M}_{2}}$ and therefore the large *M*_1_ estimate in (5) differs from the Marčenko-Pastur prediction. This formula (5) ultimately enables a more accurate description of experimental data.

**Fig. 3:**
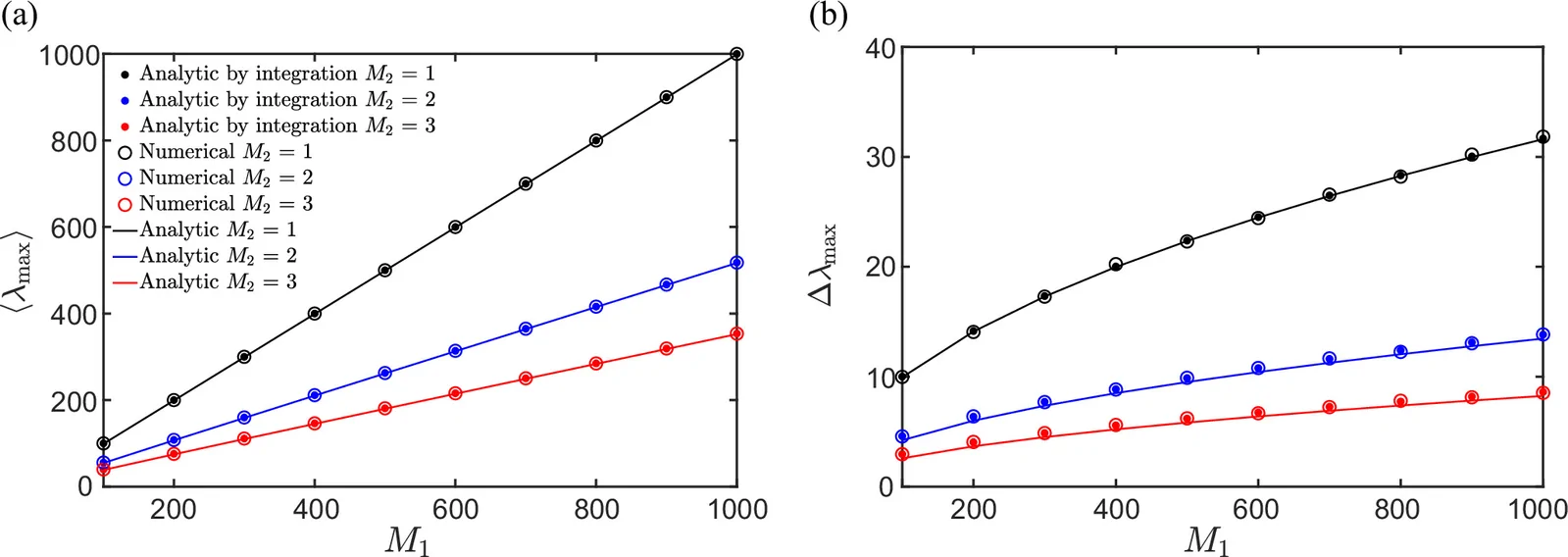
Mean and standard deviation of the largest eigenvalue in the Laguerre–Wishart ensemble. Panels **(a)** and **(b)** show respectively the mean and the standard deviation of the largest eigenvalue, ${\lambda }_{\max }$ (i.e., the enhancement factor), as a function of the number of input channels *M*_1_, for different numbers of target channels *M*_2_ = 1, 2, 3 (indicated by color). Open circles denote numerical results obtained from Laguerre-Wishart (LW) matrices, while solid circles correspond to the exact analytical predictions derived from the cumulative distribution function of ${\lambda }_{\max }$ [Eq. (4)]. Solid lines show the asymptotic large-*M*_1_ results [Eqs. (5, 6)], demonstrating excellent agreement between numerical, exact, and asymptotic results.

Similarly, by exploiting the convergence of the complex LW ensemble to the GUE in the limit *M*_1_ ≫ *M*_2_, one can obtain the behavior of the standard deviation $\Delta {\lambda }_{\max }$ in this limit. One finds indeed

6 $$\Delta {\lambda }_{\max }\approx \frac{\sqrt{2{M}_{1}}}{{M}_{2}}\Delta {\mu }_{\max }\,$$

where $\Delta {\mu }_{\max }$, which depends only on *M*_2_, is the standard deviation of the largest eigenvalue of a GUE matrix of size *M*_2_ × *M*_2_. It can be computed explicitly for the first few values of *M*_2_ (see Supplementary Note 1 for further details). These asymptotic behaviors for the mean (5) and the standard deviation (6) are compared to numerical and exact calculations in Fig. 3, showing good agreement.

We conclude this subsection by noting that in the opposite limit, when both *M*_1_ and *M*_2_ are large with a fixed ratio, the eigenvalue density converges to the Marčenko–Pastur law, supported on [*x*_−_, *x*_+_] with ${x}_{\pm }={(\sqrt{{M}_{1}/{M}_{2}}\pm 1)}^{2}$. In this limit, ${\lambda }_{\max }$ concentrates near *x*_+_, with fluctuations of order $\Delta {\lambda }_{\max } \sim {M}_{1}^{1/2}{M}_{2}^{-7/6}$, with $\Delta {\mu }_{\max } \sim {M}_{2}^{-1/6}$, governed by the celebrated Tracy–Widom (TW) distribution ^82–84^ (for a review see^85^), recovering the approximation used in Eq. (1). The familiar Tracy–Widom scaling $\Delta {\lambda }_{\max } \sim {M}^{-2/3}$ is recovered as the special case *M*_1_ = *M*_2_ = *M*. The prefactor governing the scaling of $\Delta {\lambda }_{\max }$ can be expressed in terms of the standard deviation of the Tracy-Widom distribution. Although no elementary closed-form expression is known for this constant, its value can be accurately evaluated numerically. This formula (4) thus provides both the exact finite-*M*_2_ statistics of ${\lambda }_{\max }$ and the asymptotic large-*M*_1_, *M*_2_ behavior, bridging theoretical insight and experimental relevance. Extreme eigenvalue statistics in the TW regime have been observed experimentally in other physically distinct systems in the large-size limit, such as interface growth in nematic liquid crystals ^86^, coupled lasers ^87^, and dissipative many-particle systems ^88^. In contrast, our experiments probe the largest eigenvalue of finite-size LW matrices in a wavefront-shaping setting, where the distribution generically deviates from TW statistics.

### The role of long-range mesoscopic correlations in enhancement-factor fluctuations

**Fig. 4:**
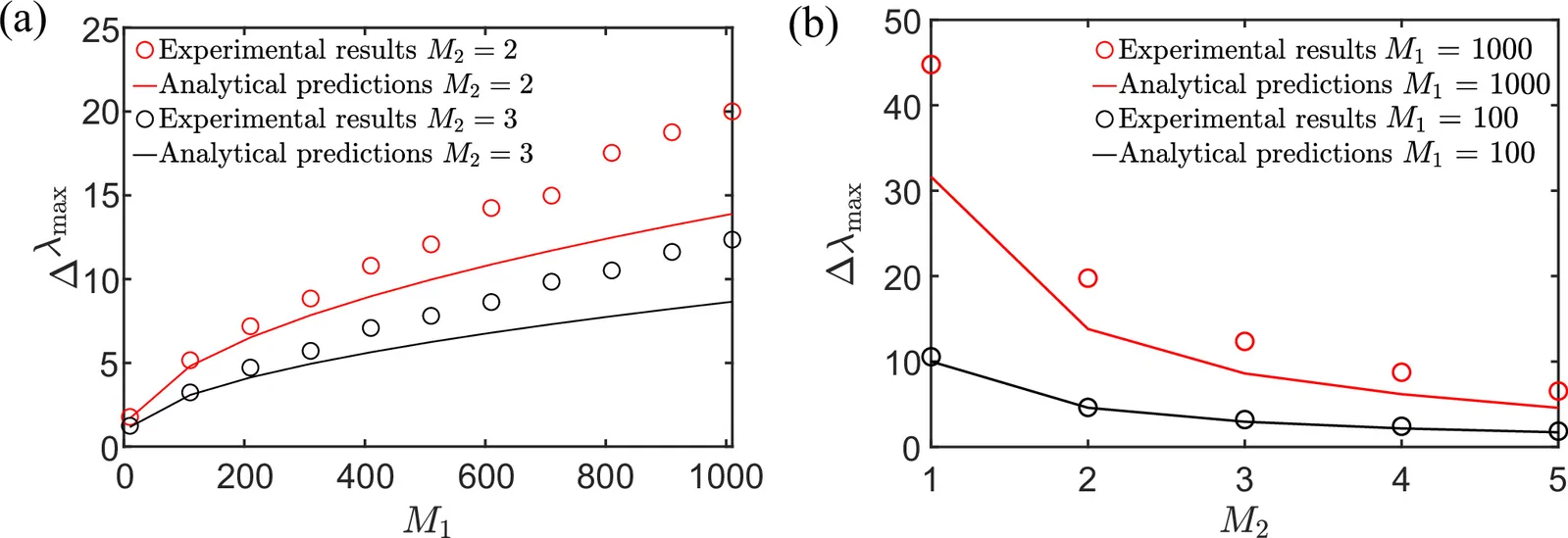
Experimental enhancement-factor fluctuations and Laguerre–Wishart predictions. Fluctuations of the enhancement factor, quantified by the standard deviation $\Delta {\lambda }_{\max }\equiv \sqrt{\langle {\lambda }_{\max }^{2}\rangle -{\langle {\lambda }_{\max }\rangle }^{2}}$, are shown versus the number of input channels *M*_1_ at fixed *M*_2_ = 2 and *M*_2_ = 3 (**a**) and versus the number of output channels *M*_2_ at fixed *M*_1_ = 100 and *M*_1_ = 1000 (**b**). The LW-ensemble theory (solid lines) agrees well with the experiments (open circles) up to *M*_1_ ≈ 100 in (**a**) and for all *M*_2_ at fixed *M*_1_ = 100 in (**b**). For *M*_1_ > 100, the experiments exhibit larger fluctuations than predicted (a,b), indicating systematic deviations from the correlation-free LW model.

Our analytical theory provides the full probability distribution of the largest eigenvalue and thus predicts its fluctuations through the standard deviation $\Delta {\lambda }_{\max }$. In Fig. 4a, b, we plot the experimentally measured $\Delta {\lambda }_{\max }$ as functions of *M*_1_ and *M*_2_, together with the correlation-free LW-based predictions. For small input-channel numbers, *M*_1_ ≲ 100, the theory reproduces the experimental fluctuations. For larger *M*_1_, however, the theory increasingly underestimates the fluctuations, consistent with the broader experimental ${P}_{\max }({\lambda }_{\max })$ compared to the LW prediction (Fig. 2c).

The LW-based framework is built on correlation-free random matrices. In contrast, it is well established that the transmission matrix of a disordered medium exhibits long-range mesoscopic correlations ^54–62^. The systematic excess of the experimental fluctuations over the LW prediction in Fig. 4a, b, therefore naturally raises the question of whether the observed fluctuations of the largest eigenvalue (i.e., the enhancement factor) in wavefront-shaping focusing experiments originate from such long-range correlations.

To address this question and systematically assess the role of long-range correlations—which are known to increase with sample thickness ^17,54–56^—we performed numerical simulations for slabs of varying thicknesses. Specifically, we computed the field transmission matrix *t* of two-dimensional (2D) scattering slabs with normalized thicknesses *k*_0_*L* = 1, 50, 200 and normalized transport mean free path *k*_0_*l*_*t*_ = 20.6 using a recursive Green’s function algorithm ^89,90^, where *k*_0_ = 2*π*/*λ*_0_ denotes the wavenumber corresponding to the wavelength *λ*_0_.

For each slab thickness, we systematically obtained the mean largest eigenvalue $\langle {\lambda }_{\max }\rangle$—averaged over different focusing target positions—and its standard deviation $\Delta {\lambda }_{\max }$ for sub-transmission matrices of various sizes *M*_2_ × *M*_1_ (see Supplementary Note 2).

**Fig. 5:**
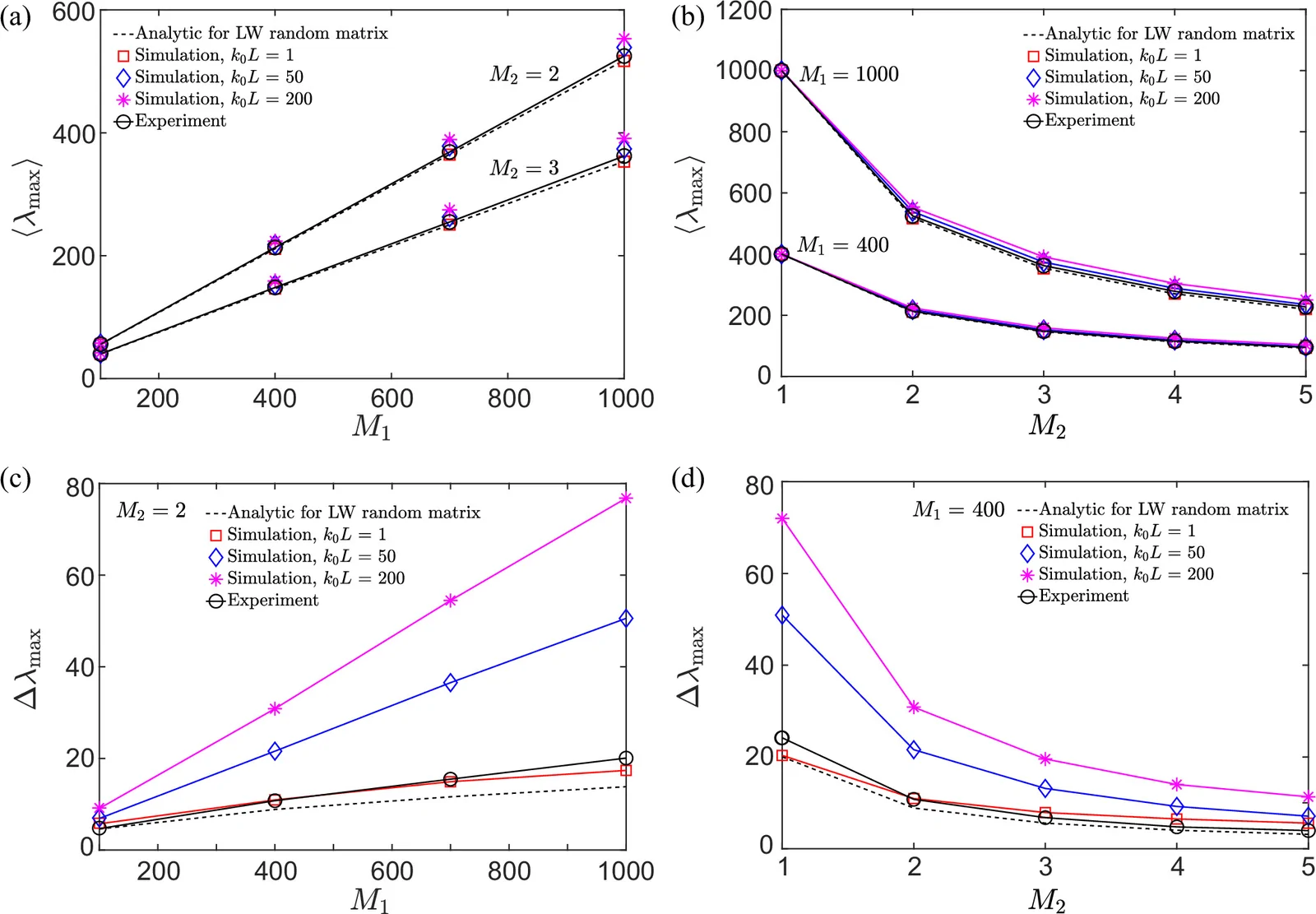
Mean enhancement factor and giant fluctuations in the presence of long-range mesoscopic correlations. The mean enhancement factor $\langle {\lambda }_{\max }\rangle$ versus the number of input channels *M*_1_ at fixed *M*_2_ = 2 and *M*_2_ = 3 (**a**) and versus the number of output channels *M*_2_ at fixed *M*_1_ = 400 and *M*_1_ = 1000 **(b)** are shown for the analytic LW-ensemble theory (dashed black line), experiments (black open circles), and numerical simulations (red open squares for *k*_0_*L* = 1, blue open diamonds for *k*_0_*L* = 50, and magenta asterisks for *k*_0_*L* = 200, all with normalized transport mean free path *k*_0_*l*_*t*_ = 20.6). All results agree across *M*_1_ and *M*_2_, consistent with the mean enhancement factor being insensitive to long-range mesoscopic correlations. Fluctuations, quantified by the standard deviation $\Delta {\lambda }_{\max }\equiv \sqrt{\langle {\lambda }_{\max }^{2}\rangle -{\langle {\lambda }_{\max }\rangle }^{2}}$ versus the number of input channels *M*_1_ at fixed *M*_2_ = 2 (**c**), and versus the number of output channels *M*_2_ at fixed *M*_1_ = 400 (**d**), exhibit a slight experimental excess and a pronounced excess in simulations, which grows with slab thickness. These giant fluctuations in thick slabs reveal long-range mesoscopic correlations beyond the correlation-free LW model.

In Fig. 5, we show the numerical, analytical, and experimental results for both the mean and the standard deviation of the largest eigenvalue. As seen in Fig. 5a, b, the numerical mean largest eigenvalue $\langle {\lambda }_{\max }\rangle$ (i.e., the enhancement factor *η*) scales with the number of input channels *M*_1_ and the number of output channels *M*_2_ in excellent agreement with the LW-random-matrix-based analytical results and with the experimental results, for all slab thicknesses. This behavior is consistent with the mean enhancement factor being insensitive to long-range mesoscopic correlations ^10,17^ and, consequently, to the slab thickness.

The situation is different for the fluctuations. In Fig. 5c, d, we observe a clear increase in the standard deviation $\Delta {\lambda }_{\max }$ of the largest eigenvalues as the slab becomes thicker in the simulations, which is a clear signature of long-range mesoscopic correlations governing these fluctuations. Moreover, our simulation results show that the observed fluctuations can be several times larger than the correlation-free LW prediction, thus indicating that long-range mesoscopic correlations can give rise to giant fluctuations of the enhancement factor, far beyond the finite-size random-matrix baseline.

Remarkably, even for extremely thin slabs (*L* ≪ *l*_*t*_), the fluctuations already start to deviate from the correlation-free prediction as *M*_1_ increases (Fig. 5c), and the experimental data show the same trend. One might expect even larger deviations in the experiment, since the sample is much thicker than a transport mean free path (*L* ≫ *l*_*t*_). However, the observable strength of long-range correlations decreases with the input-control ratio *M*_1_/*g*, where *g* is the dimensionless conductance or number of open channels ^15,17,70^. Because our experimental slab is three-dimensional (3D), *g* is much larger than in the 2D simulations, and therefore the effective input-control ratio is substantially smaller. This reduced control weakens the impact of long-range correlations on $\Delta {\lambda }_{\max }$ in the experiment, explaining the quantitative differences while preserving the qualitative agreement.

Traditionally, long-range correlations are revealed through the variance of the eigenvalue density of *t*^†^*t* or through shifts in $\langle {\lambda }_{\max }\rangle$, but this requires measuring large transmission matrices with *M*_1_ ≳ 2000 inputs and *M*_2_ ≳ 3000 outputs through very thick samples (≈50*l*_*t*_) ^17^. In contrast, we show here that long-range correlations can be detected from much smaller datasets—using submatrices with only *M*_1_ ≲200 and even a single output channel (*M*_2_ = 1)—and through samples as thin as ≈10*l*_*t*_. This is possible because the LW-based analytical framework provides exact predictions in the absence of correlations, allowing systematic excess fluctuations beyond the correlation-free baseline to serve as a sensitive signature of long-range mesoscopic correlations.

## Discussion

In conclusion, our work establishes the statistical foundations of the enhancement factor, a key measure of coherent control of light transport through complex scattering media with implications for biomedical imaging, optical metrology, secure communications, optical trapping, and quantum photonics. By combining experiments, numerical simulations, and exact random-matrix results, we determine the full probability distribution of the enhancement factor for multi-point focusing. For finite numbers of input and output channels, and within the Laguerre–Wishart (LW) random-matrix framework (i.e., in the absence of long-range mesoscopic correlations), the theory quantitatively captures both the mean and the fluctuations, going beyond the Marčenko–Pastur prediction for the mean and the Tracy–Widom law for fluctuations in the asymptotic large-channel limit. These results provide, for the first time, a comprehensive statistical description directly relevant to experimentally accessible finite-channel regimes.

Beyond applying the Laguerre–Wishart framework to enhancement-factor statistics in wavefront shaping, we demonstrate that strongly scattering media exhibit giant fluctuations arising from long-range mesoscopic correlations. While probing long-range mesoscopic correlations through the mean largest eigenvalue would normally require nearly complete transmission matrices containing 10^4^–10^6^ channels ^13,15,17,70,91^, our results show that enhancement-factor fluctuations already provide a sensitive probe when only *M*_1_ ≲ 200 input and *M*_2_ = 1 output channels are controlled. This establishes a direct connection between wavefront-shaping experiments and mesoscopic wave transport, revealing the limitations of correlation-free random-matrix predictions in the presence of long-range mesoscopic correlations.

A natural question is whether these correlation-induced deviations can instead be described within the framework of correlated Wishart ensembles ^92–95^. Such models introduce correlations through the second-order covariance structure of the Gaussian transmission matrix, $\left\langle {t}_{{j}_{1}{i}_{1}}^{*}{t}_{{i}_{2}{j}_{2}}\right\rangle$, which characterizes pairwise correlations between transmission coefficients, whereas the long-range mesoscopic correlations considered here arise from the coherent interference of multiple scattering paths and are characterized by higher-order four-field connected correlation functions (i.e., fourth-order cumulants), ${\left\langle {t}_{{j}_{1}{i}_{1}}^{*}{t}_{{i}_{2}{j}_{2}}{t}_{{j}_{3}{i}_{3}}^{*}{t}_{{i}_{4}{j}_{4}}\right\rangle }_{c}$^54–56^, rather than solely by second-order covariances. Consequently, these physical correlations cannot, in general, be represented solely through a prescribed covariance matrix for Gaussian transmission coefficients. Existing theories either explicitly incorporate long-range mesoscopic correlations into random-matrix models while describing only the global eigenvalue density ^70^, or accurately reproduce the global spectrum without explicitly encoding these correlations, as in radiant-field theory ^96,97^. Establishing a predictive mapping between mesoscopic multiple-scattering theory and correlated random-matrix theory—and ultimately an accurate theory for the extreme eigenvalue statistics in the presence of long-range mesoscopic correlations—therefore remains an important open problem.

Although demonstrated here for optical wavefront shaping, the underlying mechanism relies only on multiple scattering and finite-size statistics, suggesting that the fluctuation physics uncovered in this work should extend naturally to acoustic, elastic, microwave, and matter-wave systems. More broadly, our results establish the enhancement factor not merely as a measure of focusing performance, but as a statistical observable that provides a simple and experimentally accessible probe of long-range mesoscopic correlations in complex scattering media.

## Methods

### Experimental setup and transmission-matrix measurements

We measure the field transmission matrix of a ~10 μm-thick layer of densely packed ZnO nanoparticles (with average diameter ~200 nm) deposited on a 170 μm-thick cover slip (average transmittance ~ 0.2 at *λ* = 532 nm) using a Mach-Zehnder interferometer (shown in Supplementary Fig. 3). A linearly polarized 532-nm laser beam (Coherent, Compass 215M-50 SL) is expanded, collimated, and split into two arms; in the sample arm, the beam reflects off a spatial light modulator (Hamamatsu X10468-01) and is imaged onto the pupil of a microscope objective MO_1_ (Nikon 100×, NA_in_ = 0.95) by lenses *L*_1_ (*f*_1_ = 100 mm) and *L*_2_ (*f*_2_ = 250 mm), with an iris blocking higher diffraction orders, such that each SLM segment corresponds approximately to a plane wave incident on the sample surface at a specific angle. The transmitted light is collected by an oil-immersion microscope objective MO_2_ (Edmund 100×, NA_out_ = 1.25), collimated by *L*_5_ (*f*_5_ = 200 mm) and *L*_6_ (*f*_6_ = 150 mm), and interfered with the reference beam; the interference pattern is recorded at the Fourier plane of the sample output by a CCD camera (Allied Vision Manta G-031B), after a single linear polarization selection.

The transmission matrix is measured in the Hadamard basis using four-phase-shift interferometry. A circular aperture on the SLM covers 2415 segments, from which 32 × 32 = 1024 central segments (each 9 × 9 pixels) are used as input channels, while a high-frequency grating outside this region diffracts light away. Four global phase shifts, equally spaced between 0 and 2*π*, are applied to the Hadamard patterns to extract the relative phase with the reference beam; blocking the reference allows for intensity measurements of all inputs. Combining these yields the complex transmitted field, which is detected on a 64 × 64 = 4096-pixel CCD region, giving a transmission matrix ${t}_{m{\prime} n{\prime} }$ of size 4096 × 1024. We then apply a Hadamard transform as ${t}_{m^{\prime} n}=\mathop{\sum }_{n^{\prime}=1}^{N}{t}_{m^{\prime} n^{\prime} }{H}_{n^{\prime} n}$ to convert the transmission matrix into the canonical (SLM pixel) basis *n*. Here ${H}_{n{\prime} n}$ represents the unitary Hadamard transform matrix. In our experimental setup, each output index $m{\prime}$ corresponds to a CCD camera pixel, representing a single Fourier component on the sample back surface. Since the relative phase between each output channel $m{\prime}$ is known, a two-dimensional Fourier transform can be applied to each column of the transmission matrix ${t}_{m{\prime} n}$ to obtain the transmission matrix *t*_*mn*_, where *m* denotes the position on the back surface of the sample, mapping the light field on the SLM to the outgoing field at the back surface of the sample.

After obtaining the experimental transmission matrix *t*_*mn*_, where *n* denotes the SLM segment index and *m* the position index on the sample back surface, for each choice of *M*_2_, we normalize the transmission-matrix elements such that Var(*t*_*mn*_) = 1/*M*_2_. We then construct an ensemble of randomly selected sub-transmission matrices *A* of size *M*_2_ × *M*_1_. We focus on the regime *M*_1_ ≫ *M*_2_, with ${M}_{1}\in [100,1000]\cap {\mathbb{Z}}$ and ${M}_{2}\in [1,5]\cap {\mathbb{Z}}$. Each sub-matrix *A* is formed by randomly choosing *M*_1_ input segments on the SLM and *M*_2_ output target positions in transmission. Since the mean transmitted intensity profile on the back surface of the sample is not spatially uniform due to diffusion, we restrict the rows of *A* to the central region of the diffuse spot, where the profile is approximately uniform, and select the target positions accordingly.

### Wave-propagation simulations

In our simulations, we model wave propagation through two-dimensional (2D) diffusive slabs of size *k*_0_*W* × *k*_0_*L*, where *W* and *L* denote the slab width and length, respectively. The sample is discretized on a 2D square grid with grid spacing (*λ*_0_/2*π*) × (*λ*_0_/2*π*). The dielectric constant at each grid point is defined as

7 $$\epsilon ({{\bf{r}}})={n}_{0}^{2}+\delta \epsilon ({{\bf{r}}}),$$

where *n*_0_ is the average refractive index of the medium, and *δϵ*(**r**) is a uniformly distributed random number in the range [−Σ, Σ]. The slab is embedded between two homogeneous media of refractive indices *n*_1_ and *n*_2_, and periodic boundary conditions are imposed along the transverse direction. The field transmission matrix *t*_*mn*_ at wavelength *λ*_0_ is computed by solving the scalar wave equation

8 $$\left[{\nabla }^{2}+{k}_{0}^{2}\epsilon ({{\bf{r}}})\right]\psi ({{\bf{r}}})=0,$$

with *k*_0_ = 2*π*/*λ*_0_ the wavenumber corresponding to the wavelength *λ*_0_, using the recursive Green’s function method ^89,90^.

Starting from the computed transmission matrix *t*_*mn*_, where *n* denotes the index of incident Fourier components at the input and *m* the spatial position at the output surface of the scattering slab, we construct an ensemble of focusing operators *A*^†^*A*. Here, *A* is a randomly selected sub-transmission matrix of dimension *M*_2_ × *M*_1_, constructed from the normalized transmission matrix *t*, whose elements satisfy Var(*t*_*mn*_) = 1/*M*_2_. We focus on the regime *M*_1_ ≫ *M*_2_, with ${M}_{1}\in [100,1000]\cap {\mathbb{Z}}$ and ${M}_{2}\in [1,5]\cap {\mathbb{Z}}$. Each sub-matrix *A* is formed by randomly choosing *M*_1_ input channels and *M*_2_ output target positions *m* in transmission.

For every sub-matrix size *M*_2_ × *M*_1_, we compute the eigenvalues from

9 $${A}^{{\dagger} }A{V}_{\alpha }={\lambda }_{\alpha }{V}_{\alpha },$$

and determine the probability density ${P}_{\max }({\lambda }_{\max })$, the mean $\langle {\lambda }_{\max }\rangle$, and the standard deviation

10 $$\Delta {\lambda }_{\max }=\sqrt{\langle {\lambda }_{\max }^{2}\rangle -{\langle {\lambda }_{\max }\rangle }^{2}}$$

of the largest eigenvalue. This procedure yields the statistical distribution of the enhancement factor for focusing light through a scattering slab. We apply it to transmission matrices *t*_*mn*_ of slabs with *k*_0_*W* = 6000, Σ = 0.60 (the normalized transport mean free path of *k*_0_*l*_*t*_ = 20.6), *n*_0_ = *n*_1_ = *n*_2_ = 1.5, and normalized thicknesses *k*_0_*L* ∈ {1, 50, 200}.

## Supplementary information

Supplementary Information

Transparent Peer Review file

## Data Availability

The raw transmission-matrix datasets generated in this study are not deposited in a public repository because of their large file size. These data are available from the corresponding authors upon request. Requests should be directed to either of the corresponding authors and will normally be addressed within eight weeks. There are no restrictions on who may request the data or on their use for research purposes. Once provided, the data will remain available to the requester without a time limit.
