# Supplementary material for: Largest eigenvalue statistics of wavefront shaping in complex scattering media
Source: Nat Commun. 2026 Sep 30;17:10129. doi: 10.1038/s41467-026-77933-y (PMC13627650; doi:10.1038/s41467-026-77933-y)
Supplement: Supplementary file 1 [file 41467_2026_77933_MOESM1_ESM.pdf]

Supplementary Information for "Largest eigenvalue statistics of wavefront shaping in complex scattering media"

Grégory Schehr[1,*] and Hasan Yılmaz[2,†]

[1]Sorbonne Université, Laboratoire de Physique Théorique et Hautes Energies
CNRS UMR 7589, 4 Place Jussieu, 75252 Paris Cedex 05, France
[2]Department of Electrical and Computer Engineering,
Saint Louis University, St. Louis, Missouri 63103, USA

This document provides supplementary information for "Largest eigenvalue statistics of wavefront shaping in complex scattering media." Supplementary Note 1 provides additional details of the analytical approach based on random matrix theory. Supplementary Notes 2–4 present additional numerical and experimental results supporting the main text.

Supplementary Note 1. Analytical approach via random matrix theory

In this section, we provide some details about the analytical results provided in the main text. Our starting point is the joint probability distribution function (JPDF) of the M_2 real and nonzero eigenvalues given for the focusing operator $A^\dagger A$ in Eq. (2) of the main text, which we recall here

$$P(\lambda_1, \ldots, \lambda_{M_2}) = \frac{1}{Z} \prod_{i=1}^{M_2} \lambda_i^\nu \, e^{-M_2 \lambda_i} \prod_{i<j} (\lambda_i - \lambda_j)^2 \;, \tag{S1}$$

where $\nu = M_1 - M_2 > 0$. The normalization factor can be explicitly computed, leading to

$$Z = M_2^{-M_1 M_2} \prod_{k=0}^{M_2-1} [(k+1)!(k+\nu)!] \;. \tag{S2}$$

A. Average density

The density of nonzero eigenvalues $\tilde{\rho}(\lambda)$ is defined as

$$\tilde{\rho}(\lambda) = \sum_{i=1}^{M_2} \langle \delta(\lambda - \lambda_i) \rangle \tag{S3}$$

where $\delta(x)$ denotes the Dirac delta function and with the notation $\langle \cdots \rangle$ referring to an average over the random eigenvalues $\lambda_1, \cdots, \lambda_{M_2}$ distributed according to the JPDF in (S1). By inserting this explicit expression for the JPDF in Eq. (S3) and using its invariance under permutation of the λ_i's we see that computing $\tilde{\rho}(\lambda)$ amounts to perform the following multiple integral

$$\tilde{\rho}(\lambda) = M_2 \int_0^\infty d\lambda_2 \cdots \int_0^\infty d\lambda_{M_2} P(\lambda, \lambda_2, \cdots, \lambda_{M_2}) \;. \tag{S4}$$

It turns out that this multiple integral can be performed explicitly using standard tools in random matrix theory (RMT), e.g., orthogonal polynomials, leading to the result given in Eq. (3) of the main text. In fact, in this formula (3), the sum over k can be explicitly computed using the Christoffel-Darboux relation

$$\tilde{\rho}(\lambda) = \frac{M_2! M_2^{\nu+1} \lambda^\nu e^{-M_2 \lambda}}{(M_2 + \nu - 1)!} \Big[L_{M_2-1}^{(\nu)}(M_2\lambda) L_{M_2-1}^{(\nu+1)}(M_2\lambda) - L_{M_2}^{(\nu)}(M_2\lambda) L_{M_2-2}^{(\nu+1)}(M_2\lambda) \Big] \;, \tag{S5}$$

* schehr@lpthe.jussieu.fr
† hasan.yilmaz@slu.edu

where $L_k^{(\nu)}(\lambda)$ is the generalized Laguerre polynomial of index ν and degree k, with the convention $L_{-1}^{\nu+1}(x) = 0$ for all x. This density has a nontrivial structure exhibiting M_2 local maxima, corresponding to the typical location of the M_2 nonzero eigenvalues of the Anti-Wishart matrix $A^\dagger A$. Its asymptotic behaviors for small and large λ are given by

$$\tilde{\rho}(\lambda) \approx \begin{cases} C_0\, \lambda^{\nu} \quad , & \lambda \to 0 \\ \\ C_\infty\, \lambda^{\nu+2M_2-2}\, e^{-M_2\,\lambda} \quad , & \lambda \to \infty\,, \end{cases} \tag{S6}$$

where the amplitudes C_0 and C_∞ are given by

$$C_0 = \frac{M_2^{1+\nu}(M_2+\nu)!}{(M_2-1)!\nu!(\nu+1)!}\,, \tag{S7}$$

$$C_\infty = \frac{M_2^{\nu+2M_2-1}}{(M_2+\nu-1)!(M_2-1)!}\,. \tag{S8}$$

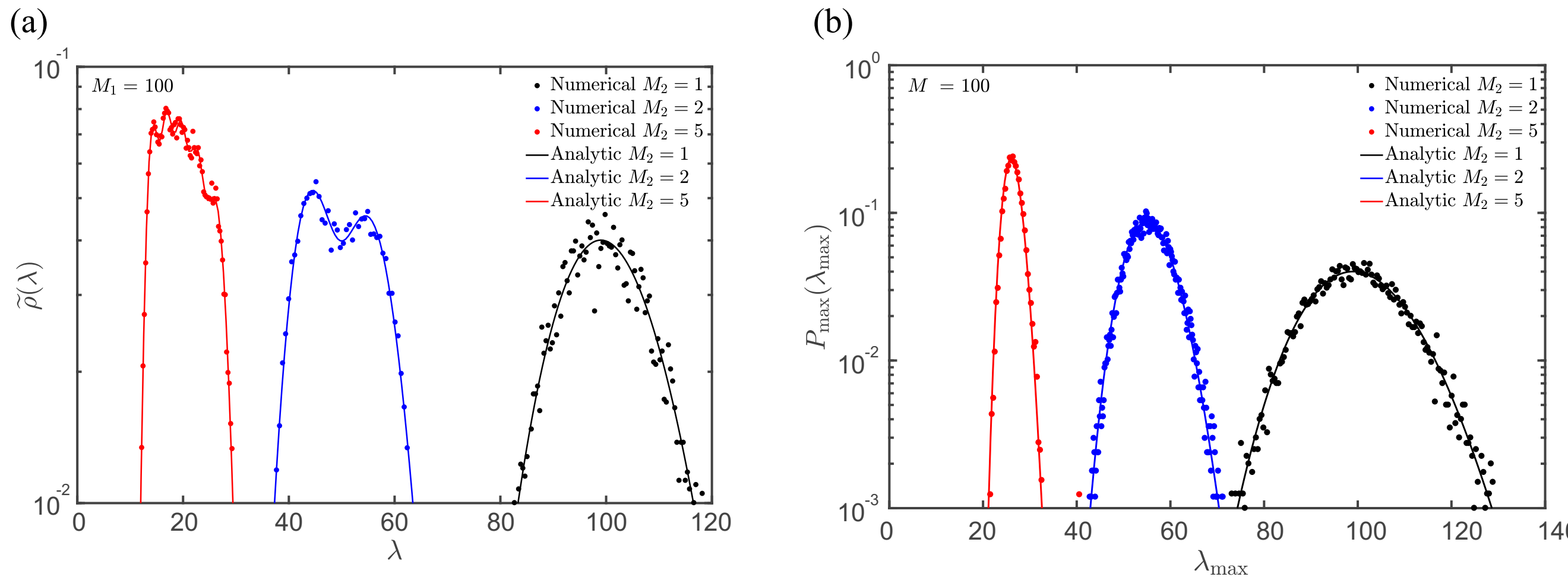

Supplementary Fig. 1. (a) Eigenvalue distributions $\widetilde{\rho}(\lambda)$ and (b) largest-eigenvalue distributions $P_{\max}(\lambda_{\max})$ for $M_1 = 100$ and $M_2 = 1, 2, 5$. The LW–random-matrix-based predictions and the numerical Wishart-matrix results coincide remarkably well for both $\widetilde{\rho}(\lambda)$ given in (S5) and $P_{\max}(\lambda_{\max}) = Q'_{\max}(\lambda_{\max})$ given in (S12).

A plot of the density given $\tilde{\rho}(\lambda)$ in Eq. (S5) vs λ is shown in Supplementary Fig. 1 (a) for $M_1 = 100$ and different values of $M_2 = 1, 2, 5$ along with numerical results obtained by direct diagonalization of LW matrices, demonstrating a good agreement. For $M_2 = 2$ and $M_2 = 5$, we observe two and five local maxima, respectively. These peaks reflect the typical positions of the corresponding nonzero eigenvalues of $A^\dagger A$. Because of eigenvalue repulsion, these eigenvalues form a relatively rigid spectrum: their individual values fluctuate, but their locations remain sharply concentrated around well-defined mean positions. Consequently, the probability density develops distinct peaks near the characteristic positions of the first, second, third, and so on, eigenvalues. As M_2 becomes larger, these individual peaks gradually merge, and in the asymptotic limit of large M_2 (and M_1), the spectrum converges to the Marčenko-Pastur distribution.

B. Cumulative distribution function of $\lambda_{\max}$

As explained in the main text, the cumulative distribution function (CDF) of $Q_{\max}(x) = \text{Prob}(\lambda_{\max} \leq x) = \text{Prob}(\lambda_1 \leq x, \cdots, \lambda_{M_2} \leq x)$ can be written as the following multiple integral

$$Q_{\max}(x) = \int_0^x d\lambda_1 \cdots \int_0^x d\lambda_{M_2} P(\lambda_1, \ldots, \lambda_{M_2})\,. \tag{S9}$$

This multiple integral can be performed explicitly using the standard Cauchy-Binet formula [1], leading to

$$Q_{\max}(x) = \frac{M_2!}{Z}\, \det \mathbb{M}, \quad \mathbb{M}_{i,j} = \int_0^x \lambda^{i+j-2+\nu} e^{-M_2 \lambda}\, d\lambda\,, \tag{S10}$$

with $i,j = 1,\cdots,M_2$. We recall that Z is given in Eq. (S2). By expressing the integral over λ that enters the definition of the matrix elements $\mathbb{M}_{i,j}$ in (S10) in terms of the incomplete gamma function $\gamma(s,z) = \int_0^z t^{s-1}e^{-t}\,dt$ and performing standard manipulations of the determinant yields the expression given in Eq. (4) in the main text. In fact, since $\nu = M_1 - M_2 > 0$ is an integer, this integral over λ can be performed explicitly, leading to

$$\begin{aligned}\mathbb{M}_{i,j} &= \int_0^x \lambda^{i+j-2+\nu} e^{-M_2\lambda}\,d\lambda \\ &= \frac{(i+j-2+\nu)!}{M_2^{i+j-1+\nu}}\left(1 - e^{-M_2 x}\sum_{k=0}^{i+j-2+\nu}\frac{(M_2 x)^k}{k!}\right),\end{aligned} \tag{S11}$$

which holds provided i,j and $\nu = M_1 - M_2$ are integers (which is indeed the case here). Injecting this in Eq. (S10), one finds the fairly explicit expression

$$Q_{\max}(x) = \frac{M_2!}{\prod_{k=0}^{M_2-1}(k+1)!\,(k+\nu)!}\det_{1\le i,j\le M_2}\left[(i+j-2+\nu)!\left(1-e^{-M_2x}\sum_{k=0}^{i+j-2+\nu}\frac{(M_2x)^k}{k!}\right),\right] \quad,\ \nu = M_1-M_2\ . \tag{S12}$$

Note that for $M_2 = 1$ one finds the simple result $P_{\max}(x) = dQ_{\max}(x)/dx = x^{M_1-1}\,e^{-x}/(M_1-1)!$, as in [2], but already for $M_2 = 2$ the expression is much more complicated.

From this explicit expression, one can obtain the asymptotic behaviors of $Q_{\max(x)}$. As $x \to 0$ it behaves as $Q_{\max}(x) \propto x^{M_1M_2}$ while $1 - Q_{\max}(x) \to q_{M_1,M_2}(x)e^{-M_2x}$ as $x \to \infty$, where $q_{M_1,M_2}(x)$ is a polynomial of order $M_1 + M_2 - 2$.

A plot of the density given $P_{\max}(\lambda_{\max}) = Q'_{\max}(\lambda_{\max})$ from Eq. (S12) vs $\lambda_{\max}$ is shown in Supplementary Fig. 1 (b) for $M_1 = 100$ and different values of $M_2 = 1, 2, 5$ along with numerical results obtained by direct diagonalization of LW matrices, demonstrating a good agreement.

C. The limit of large $M_1 \gg M_2$

In the limit where $M_1 \gg M_2$ one can show that the complex Wishart ensemble described by (S1) converges, after centering around M_1/M_2 and rescaling by $M_2/\sqrt{2M_1}$, to the Gaussian Unitary Ensemble (GUE) that describes Hermitian matrices with independent (complex) Gaussian entries. Consequently for large $M_1 \gg M_2$ one finds

$$\langle\lambda_{\max}\rangle \approx \frac{1}{M_2}\left(M_1 + \sqrt{2\,M_1}\,\langle\mu_{\max}\rangle\right)\ , \tag{S13}$$

where $\mu_{\max}$ denotes the largest eigenvalue of a GUE matrix of size $M_2 \times M_2$. Its CDF $\mathcal{Q}_{\max}(x) = \text{Prob.}(\mu_{\max} \le x)$ can be computed as explained above, but transposed to the case of GUE. It reads

$$\mathcal{Q}_{\max}(x) = \frac{1}{\tilde{Z}}\det\widetilde{\mathbb{M}}\ ,\ \widetilde{\mathbb{M}}_{i,j} = \int_{-\infty}^x \mu^{i+j-2}\,e^{-\mu^2}\,d\mu\ , \tag{S14}$$

with $\tilde{Z} = \det_{i,j=1}^{M_2}\int_{-\infty}^{\infty}\mu^{i+j-2}\,e^{-\mu^2}\,d\mu$. From this expression for the CDF in (S14) one can obtain the average $\langle\mu_{\max}\rangle = \int_{-\infty}^{\infty} x\mathcal{Q}'_{\max}(x)dx$ yielding in particular

$$\langle\mu_{\max}\rangle = \begin{cases} 0\ , & M_2 = 1\ , \\ \sqrt{\dfrac{2}{\pi}}\ , & M_2 = 2\ , \\ \dfrac{27}{8\sqrt{2\pi}}\ , & M_2 = 3\ , \end{cases} \tag{S15}$$

as given in the text.

In this limit $M_1 \gg M_2$ one can also compute the behavior of the standard deviation. Indeed from the convergence of the complex LW ensemble to the GUE (of size $M_2 \times M_2$), one has the identity in law

$$\lambda_{\max} \approx \frac{1}{M_2}(M_1 + \sqrt{2M_1}\mu_{\max})\ , \tag{S16}$$

where, as before, $\mu_{\max}$, which depends only M_2 and not on M_1, denotes the largest eigenvalue of a GUE matrix of size $M_2 \times M_2$. This identity (S16) allows to obtain straightforwardly the large M_1 behavior of the standard deviation $\Delta\lambda_{\max}$ as given in the text, namely

$$\Delta\lambda_{\max} \approx \frac{\sqrt{2M_1}}{M_2}\Delta\mu_{\max} \;, \tag{S17}$$

where $\Delta\mu_{\max}$, which depends only on M_2, is the standard deviation of the largest eigenvalue of a GUE matrix of size $M_2 \times M_2$. It can be computed from the CDF of $\mu_{\max}$ given in Eq. (S14). In particular, one gets

$$(\Delta\mu_{\max})^2 = \begin{cases} \dfrac{1}{2} \;, & M_2 = 1 \;, \\[2ex] 1 - \dfrac{2}{\pi} \;, & M_2 = 2 \;, \\[2ex] \dfrac{3}{2} + \dfrac{9}{128\pi}(16\sqrt{3} - 81) \;, & M_2 = 3 \;. \end{cases} \tag{S18}$$

These asymptotic behaviors in Eqs. (S13)-(S15) and (S17)-(S18) are compared to numerical and exact results in Fig. 3 of the main text, showing an excellent agreement between the different estimations.

Supplementary Note 2. Eigenvalue probability densities from wave-propagation simulations

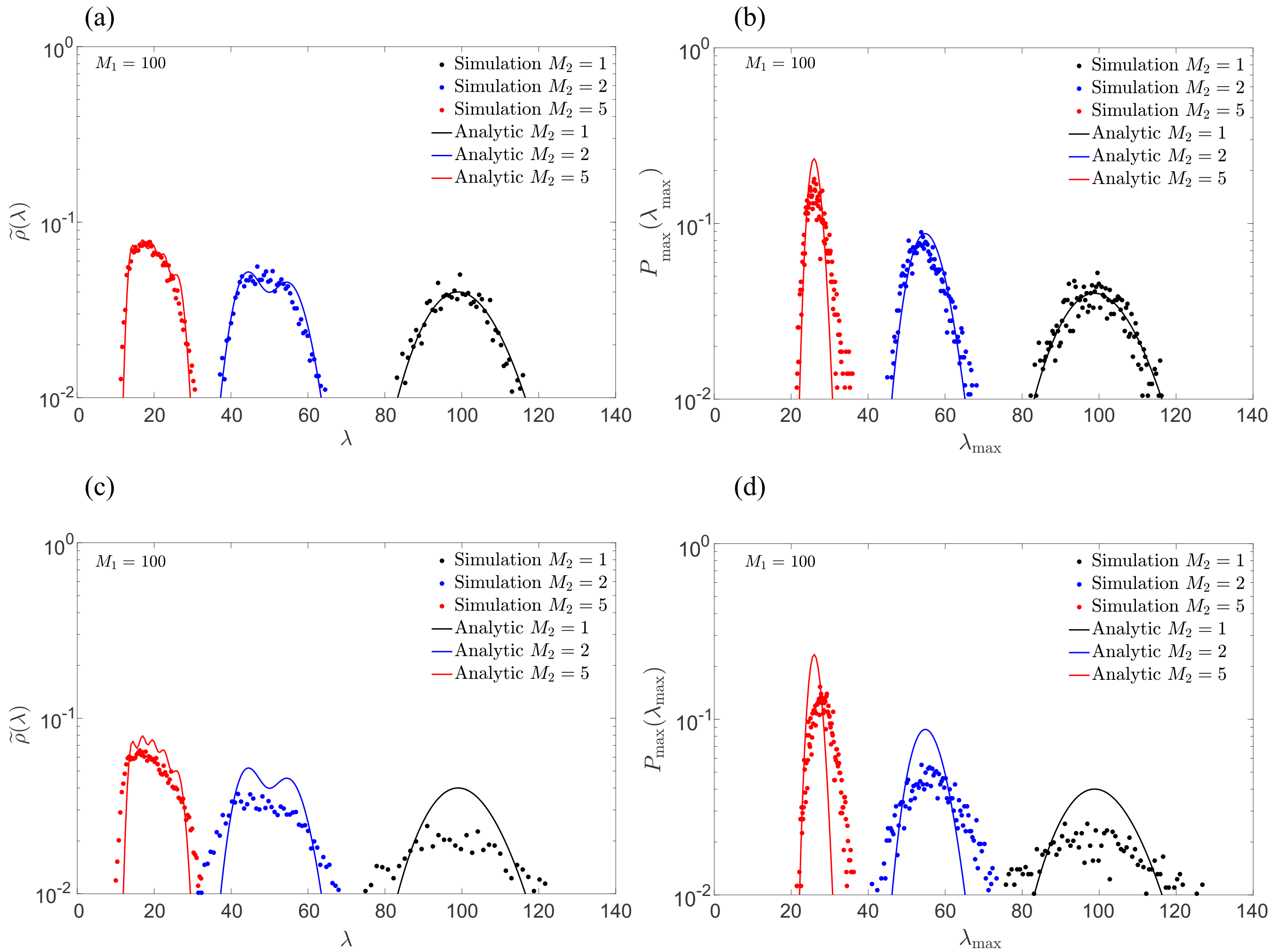

Supplementary Fig. 2. (a) Eigenvalue distributions $\widetilde{\rho}(\lambda)$ and (b) largest eigenvalue distributions $P_{\max}(\lambda_{\max})$ for $M_1 = 100$ and $M_2 = 1, 2, 5$ for a diffusive slab with normalized thickness $k_0 L = 1$ and normalized transport mean free path $k_0 l_t = 20.6$. For this case, the LW–random-matrix predictions for $\widetilde{\rho}(\lambda)$ [Eq. (S5)] and for $P_{\max}(\lambda_{\max}) = Q'_{\max}(\lambda_{\max})$ [Eq. (S12)] are in good agreement with the wave-propagation simulations. In contrast, for a thicker slab with $k_0 L = 200$ and $k_0 l_t = 20.6$, we find clear deviations between the LW–based predictions and the simulations for both $\widetilde{\rho}(\lambda)$ (c) and $P_{\max}(\lambda_{\max})$ (d).

Supplementary Fig. 2 shows the eigenvalue distributions $\widetilde{\rho}(\lambda)$ and the largest eigenvalue distributions $P_{\max}(\lambda_{\max})$ obtained from LW–random-matrix theory and from wave-propagation simulations for diffusive slabs with $M_1 = 100$ and $M_2 = 1, 2, 5$. For the thin slab with $k_0 L = 1$ and $k_0 l_t = 20.6$ [panels (a,b)], the LW predictions accurately reproduce both the bulk eigenvalue density and the statistics of $\lambda_{\max}$, indicating that long-range mesoscopic correlations are negligible. In contrast, for the thicker scattering slab with $k_0 L = 200$ and $k_0 l_t = 20.6$ [panels (c,d)], clear, systematic deviations emerge in both $\widetilde{\rho}(\lambda)$ and $P_{\max}(\lambda_{\max})$. These deviations signal the breakdown of the uncorrelated-random-matrix assumption and reflect the growing impact of long-range mesoscopic correlations, which reshape both the eigenvalue distribution and the distribution of the largest eigenvalues.

Supplementary Note 3. Experimental setup and eigenvalue probability densities

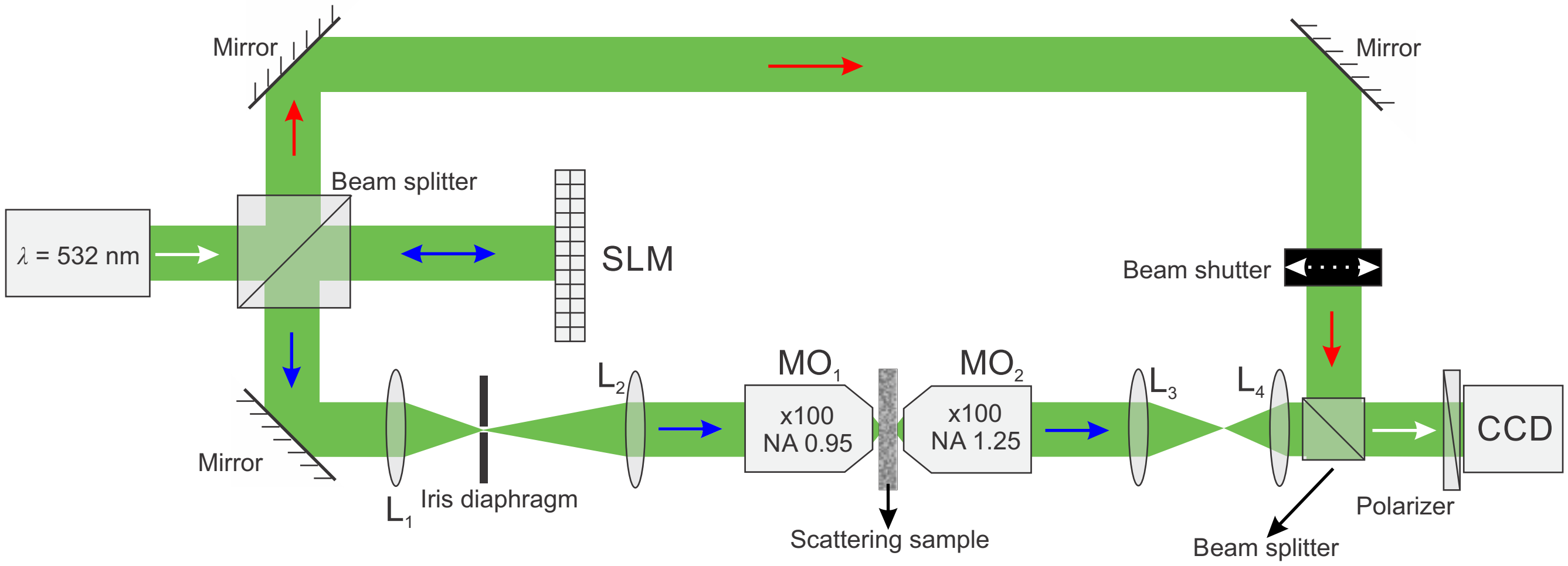

Supplementary Fig. 3. Schematic of the interferometric setup. A reflective phase-only spatial light modulator (SLM) shapes the phase of a monochromatic laser beam ($\lambda_0 = 532$ nm). Using the SLM and a CCD camera, the field transmission matrix of the scattering sample is measured in k-space. $MO_{1,2}$: microscope objectives; L_{1-4}: lenses. Reproduced from Ref. [3] under the terms of the CC BY 4.0 license.

The experimental setup used to measure the transmission matrix of the diffusive sample is shown in Supplementary Fig. 3. Supplementary Fig. 4 presents the eigenvalue densities $\widetilde{\rho}(\lambda)$ and largest eigenvalue distributions $P_{\max}(\lambda_{\max})$ obtained from LW–random-matrix theory and from experiment. For $M_1 = 110$ [panels (a,b)], the LW predictions faithfully capture both the bulk eigenvalue density and the statistics of $\lambda_{\max}$, indicating that long-range mesoscopic correlations are negligible. In contrast, for $M_1 = 1010$ [panels (c,d)], pronounced and systematic deviations appear in both $\widetilde{\rho}(\lambda)$ and $P_{\max}(\lambda_{\max})$. These deviations mark the breakdown of the uncorrelated random-matrix description and reveal the increasing influence of long-range mesoscopic correlations, which alter both the overall eigenvalue density and the distribution of the largest eigenvalues.

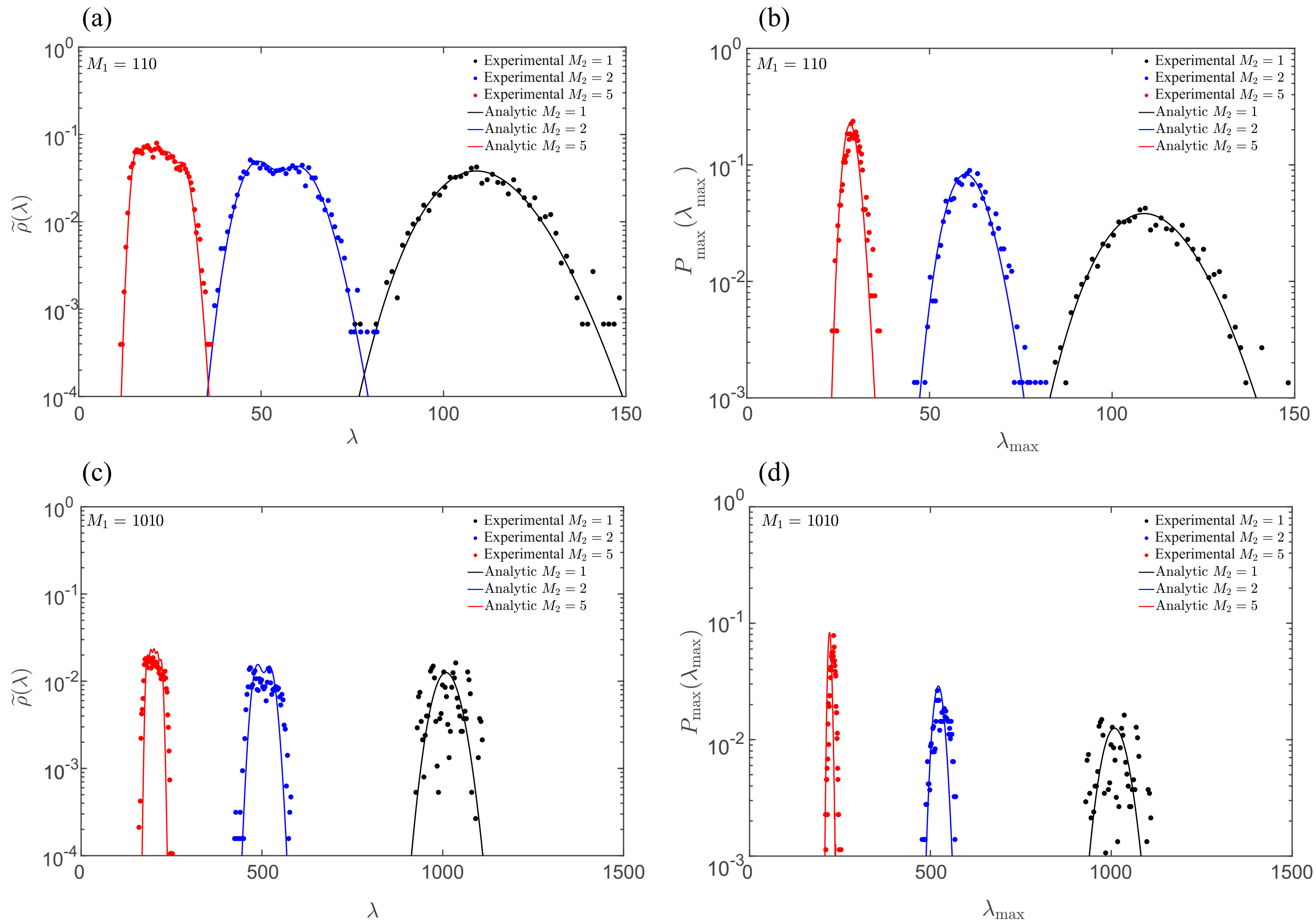

Supplementary Fig. 4. (a) Eigenvalue distributions $\widetilde{\rho}(\lambda)$ and (b) largest eigenvalue distributions $P_{\max}(\lambda_{\max})$ for $M_1 = 110$ and $M_2 = 1, 2, 5$ for the experimental diffusive slab. For this case, the LW–random-matrix predictions for $\widetilde{\rho}(\lambda)$ [Eq. (S5)] and for $P_{\max}(\lambda_{\max}) = Q'_{\max}(\lambda_{\max})$ [Eq. (S12)] are in good agreement with the experimental results. In contrast, for a larger $M_1 = 1010$, we find clear deviations between the LW–based predictions and the experiments for both $\widetilde{\rho}(\lambda)$ (c) and $P_{\max}(\lambda_{\max})$ (d).

Supplementary Note 4. Enhancement factor and intensity balance in two-target focusing

In multipoint focusing experiments, the goal is often not only to maximize the enhancement factor but also to obtain similar intensities at the target positions. It is therefore important to check whether the intensity balance between targets is correlated with the achieved enhancement. To this end, we evaluated the experimental joint probability density of the largest eigenvalue $\lambda_{\max}$ and the intensity imbalance for two-target focusing ($M_2 = 2$), defined as $R \equiv \frac{|T_1 - T_2|}{T_1 + T_2}$, where T_1 and T_2 are the intensities at the two target points (Supplementary Fig. 5). The analysis shows no discernible correlation between $\lambda_{\max}$ and R. In other words, even for large enhancement factors, one can observe either perfectly balanced intensities ($R = 0$, $T_1 = T_2$) or completely unbalanced ones ($R = 1$, $T_1 = 0$ or $T_2 = 0$) with comparable likelihood.

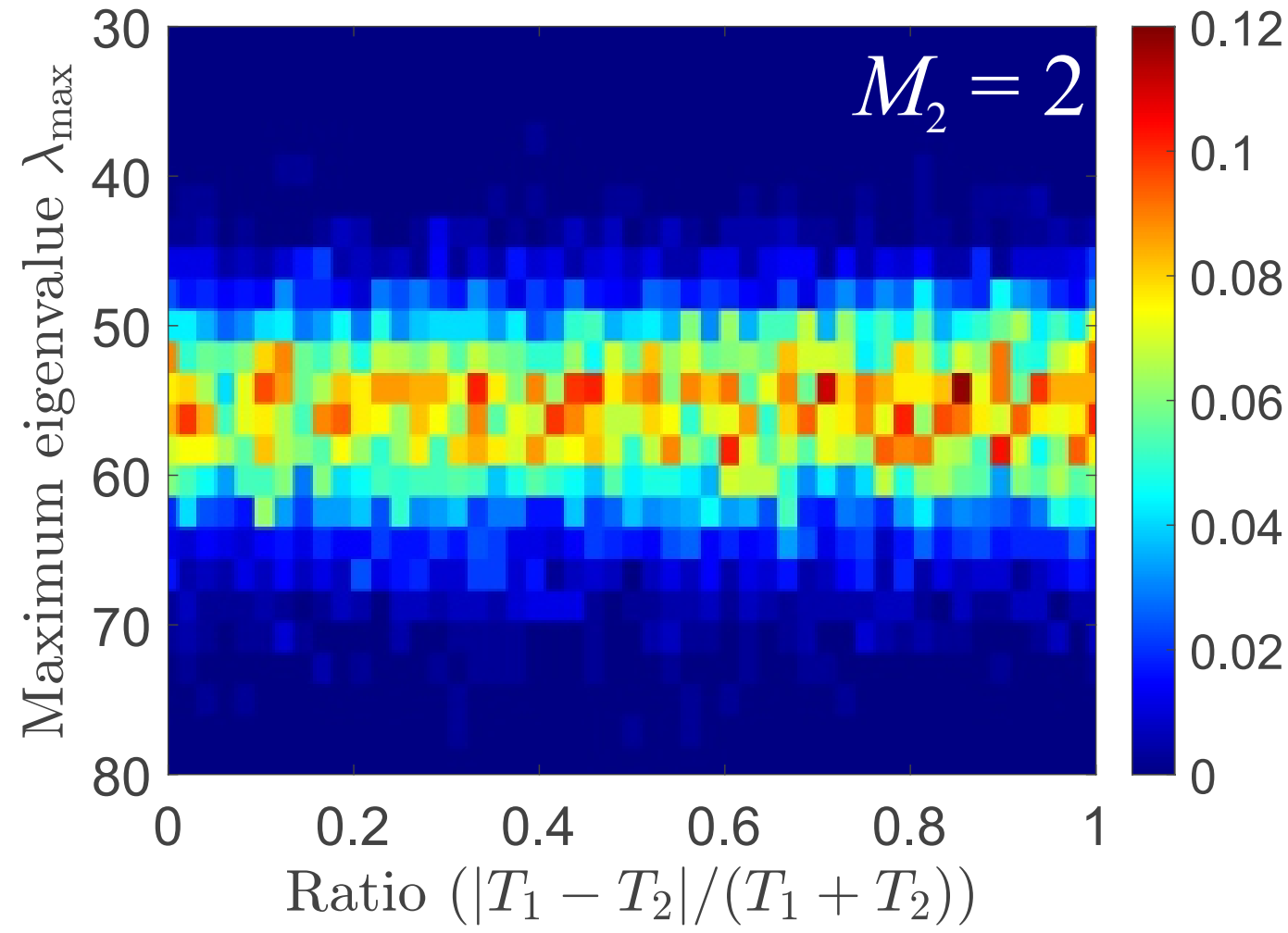

Supplementary Fig. 5. Experimental joint probability density (JPD) for two-target focusing ($M_1 = 100$, $M_2 = 2$): there is no discernible dependence between $\lambda_{\max}$ and the output intensity ratio, so high-$\lambda_{\max}$ events also occur when the two target intensities are comparable (near-equal split).

[1] P. J. Forrester, *Log-gases and random matrices (LMS-34)* (Princeton University Press, 2010).
[2] A. M. Paniagua-Diaz, W. L. Barnes, and J. Bertolotti, Wavefront shaping to improve beam quality: converting a speckle pattern into a Gaussian spot, Phys. Scr. **98**, 035516 (2023).
[3] H. Yılmaz, M. Kühmayer, C. W. Hsu, S. Rotter, and H. Cao, Customizing the angular memory effect for scattering media, Phys. Rev. X **11**, 031010 (2021).
