# Supplementary material for: Largest eigenvalue statistics of wavefront shaping in complex scattering media
Source: Nat Commun. 2026 Sep 30;17:10129. doi: 10.1038/s41467-026-77933-y (PMC13627650; doi:10.1038/s41467-026-77933-y)
Supplement: Supplementary file 2 [file 41467_2026_77933_MOESM2_ESM.pdf]

nature portfolio

Peer Review File

Largest eigenvalue statistics of wavefront shaping in complex scattering media

Corresponding Author: Professor Hasan Yılmaz

This file contains all reviewer reports in order by version, followed by all author rebuttals in order by version.

Version 0:

Reviewer comments:

Reviewer #1

(Remarks to the Author)
I read this paper with much interest and I see the potential for publication in Nature Communications. However, I feel an extensive rewriting is necessary to convey the main message in a clear and unambiguous way, and to remove parts that are quite unclear even after a few careful readings.

The main message of the paper is that experimentally accessible fluctuations of the enhancement factor already provide a sensitive probe of long-range correlations and that the enhancement factor is in fact a powerful statistical observable that encodes long-range mesoscopic correlations in complex media. This result is achieved appealing to the finite-N theory of extreme values (largest eigenvalue) for Wishart-Laguerre matrices, whose cumulative distribution can be written as a Hankel determinant thanks to the Andréief formula.

I will formulate a few comments, essentially in chronological order:

1. Stronger effort is needed in the abstract and the introduction to set the stage and describe the relevant physics. What types of 'waves' are we talking about? What is a 'complex medium'? The narrative becomes too technical too quickly. NatComm is a more generalist journal, which should be accessible to a wide array of scientists from different disciplines. Try to make sure that each sentence leads seamlessly to the next, and that all concepts/definitions/transitions are made crystal clear for any intelligent but non-specialist reader. Writing for these journals requires adopting a different perspective - your way of writing belongs more to a 'standard' physics journal audience (like PRE, PRB, or JPhysA) but will not be as attractive for this journal's readership.

2. Before eq. (1), you state that <\lambda_max> is given by the celebrated Marčenko-Pastur law. Probably you meant that the upper edge of the support of the MP law identifies the typical location of the largest eigenvalue of large Wishart matrices.

3. "We introduce a random matrix formalism" is a bit overblown. That the focusing operators constitute a Wishart ensemble is quite obvious and well-established with your assumptions - you can hardly claim that you introduced this model yourselves.

4. The issue of 'long-range (mesoscopic) correlations' is central to the paper, but it is introduced very hastily on p. 2. Please explain very clearly what these are, when they affect your WL assumptions and when they don't etc. Do not assume that the reader will be familiar with any of these concepts. More seriously, throughout the paper I have been very confused by two incarnations of the term 'correlations' which are often conflated, but (in my view) should instead be kept and discussed separately. One is the 'physical' level of mesoscopic correlations in your scattering problem (still to be defined and discussed better), the other is the 'mathematical'/probabilistic level, which treats the entries of your random matrix as independent (no 'correlations') and complex Gaussians. You seem to be arguing that the 'classical' WL ensemble (with independent entries) would correspond to the absence of physical mesoscopic correlations, but (if I understood correctly at all) this claim should be made way more evident, with a stronger justification. Conversely, it is not clear to me what the *presence* of mesoscopic correlations in your physical system would translate to in your random model. Would these be well described by a correlated Wishart ensemble, then? If yes, what should be the correlation matrix between Gaussian entries that correspond to a given level/pattern of physical correlations (however these are defined and measured)? These questions must absolutely be ironed out in detail.

5. As a further general point, the narrative becomes rapidly quite messy when it comes to theory vs numerics vs experimental results. All these are often conflated inside dense paragraphs, where the different ‘souls’ of your paper are sometimes harder to disentangle. Details about experimental setups often appear alongside numerical simulations and theoretical derivations, making some sections hard to follow. I think presentation-wise it may be more useful to clearly have separate sub-sections (Theory - Numerical Simulations - Experimental results) or - at the very least - a ‘roadmap’ section where you actually guide the reader in a schematic way on what you did for each of those components.

6. Fig. 2 (a) and (b). The ‘clear deviation’ between the Marčenko-Pastur and your LW analytical prediction is hard to assess without error bars on the experimental results. I have no problem in believing that the finite-N formulae should describe the data ‘better’, but your claim needs more evidence to be substantiated - after all, it may well be that, with the current experimental precision, the MP line does already a perfectly good job, which would severely weaken your claim.

7. I am confused again by your statement on p. 3 ‘as M1 increases, the experimental distribution broadens relative to the theoretical one’ - the way I read this sentence is: ‘there is a worrying growing discrepancy between our theory and the experiments’, which I don’t think is the message that you actually wanted to convey! What I don’t understand is: if you have a ‘better’ theory for finite M1,M2 than MP, this theory should work better across *all* values of experimentally tested M1,M2, shouldn’t it? You should not see a discrepancy between your theory and the experiments as M1 increases. I must be missing something here.

8. For fig. 2 (c) and (d), it would be helpful to provide a quantitative measure of non-Gaussianity of the blue curves. The way the results are plotted makes it unclear whether the theoretical curves are somewhat ‘skewed’ (asymmetric tails) and by how much. I am worried that a simple Gaussian fit with appropriate mean and variance would already fit the data exceedingly well - or at least as well as your theory - without the need of a very sophisticated finite-N Random Matrix theory. If this is not the case, though, it should be made quantitatively clear here.

9. Theoretical advancements. When I started reading this paper, I was excited and expected to see a somewhat ‘novel’ random matrix theory of the focusing operator and the statistics of its largest eigenvalue. I was somewhat disappointed, though, because all the theoretical finite-N results are well-known from the standard theory of unitary invariant ensembles - the determinant expression in (4) is well-known and follows from a one-liner derivation using the Andréief formula on the jpdf (2) integrated between 0 and x. Moreover, the evaluation of the determinant needs anyway to be done ‘numerically’ (or at least with the use of symbolic software) and - as the authors correctly hint - it becomes presumably quite unwieldy for increasing M2 anyway. This is not a complete deal-breaker for the publication potential of this paper, of course, but it strongly diminishes its impact: it is somewhat disheartening that the ‘waves-in-random-media’ community has not bothered thus far to go beyond Marčenko and Pastur at all, and it is a good service to that community to point out that finite-N formulae exist that do a better job - but clearly this is not ‘as strong as’ producing a better Random Matrix theory for the focusing operator, for instance incorporating the effects of mesoscopic correlations (see point 4 above), which - in the authors’ words - remains an open problem.

10. Further on my points 4. and 9. that actually nag me quite a bit. If my intuition is correct, then a ‘better’ random matrix model in presence of mesoscopic correlations should be a so-called correlated Wishart ensemble, i.e. a correlation matrix of a finite number of Gaussians whose theoretical covariance is not a multiple of the identity. Does this make sense? If so, what would be a better model for such theoretical covariance? What is known about the largest eigenvalue for finite-N of correlated Wishart matrices? A quick search returned just a few hits - not much indeed, but not zero results either… your numerical experiments (at least) or - better - your theory would greatly benefit from a comparison with the correlated Wishart largest eigenvalue results (obviously, if the mapping between experimental setting and type of theoretical correlation matrix between Gaussian elements could be established or at least estimated).

11. Another weak point of your enterprise is the fact that - while you proved that ‘long-range mesoscopic correlations’ in your physical/experimental setup can be probed by fluctuations of the enhancement factor in conjunction with your finite-M1 theory for ‘uncorrelated Gaussians’ - there doesn’t seem to be a quantitative mapping between ‘how much the discrepancy is’ in Fig. 5 (c) and (d) between the solid black line with circles and the dashed line, and the ‘amount’ of mesoscopic correlations (can this ‘amount’ be independently quantified somehow?). The argument seems to be: experiments and classical WL theory align ‘less and less’ as we tweak the experiments in such a way that we enhance these ethereal mesoscopic correlations. Ergo, this misalignment can be taken as a probe of the presence and strength of such mesoscopic correlations. But we don’t seem to have a ‘conversion table’ that tells us how much discrepancy translates into how much mesoscopic correlations - also because it is not clear to me still what these mesoscopic correlations are, and how one would measure these independently.

In summary, I see the potential for this paper to be eventually published in NatComm, if the authors can (i) robustly revise their narrative, making the paper much more accessible and streamlined, with technical details that obstruct a seamless reading relegated to a Methods section, or an appendix at the end, and (ii) clearly stating their innovative contribution in each of the components of their work (theory - numerical simulations - experiments), bearing in mind that the theoretical treatment exhibits an obvious weakness in terms of ‘novelty’ (at least to a trained RMT eye) if not reinforced by some considerations on a correlated Wishart model (or similar).

Typo: confront —> compare (p. 5)

Reviewer #2

(Remarks to the Author)
The paper develops a comprehensive analysis of the fluctuations of the enhancement factor in wave focussing experiments in a complex diffusive medium.
Theoretical part is based on assuming the entries t_{ij} of the associated transmission matrix to be i.i.d. complex gaussian variables. Such an assumption is well-justified in very open (many channel) medium in the regime of fully ergodic chaotic scattering. However in the diffusive regime it may be violated via the influence of mesoscopic long-range correlations in transmission amplitudes. The authors' approach is simple but powerful: using the above assumptions for building a suitable random matrix zero-level model, they aim at characterizing the statistics of the largest eigenvalue of the focussing operator built from $M_2\times M_1$ submatrices of the transmission matrix, with M_2 being the number of focussing targets (generally, of the order of unity),
while $M_1\gg M_2$ is the number of controlled input channels. The corresponding focussing operator is then represented by the so-called anti-Wishart random matrix of unitary symmetry class whose eigenvalues for any size are described by a determinantal process with explicitly known kernel.
Employing the standard random matrix theory (RMT) allows to represent the cumulative probability function for the largest eigenvalue as a finite-size determinant, providing a tool for its efficient evaluation. This is a relatively rare example when RMT proves to be useful beyond the large size asymptotic analysis. Having such exact representation is crucial for the ultimate goal of the paper: comparison with experimental data on focussing.
Such a comparison reveals that with increase in scattering medium widths the predictions of developed theory start showing more and more considerable deviations from the experimental data. The authors very reasonably attribute such discrepancy to the influence of mentioned long-range mesoscopic correlations neglected in the underlying assumptions on transmission matrix statistics. In this way their approach offers a sensitive test for such correlations, which otherwise are quite difficult to measure. Although none of the theoretical ingredients employed to arrive
to the conclusion is particularly new, the very idea and its skillful implementation in the paper, both theoretical and experimental, look to me certainly worthy of publications in Nature Communication.

A minor remark is due.

The authors take for granted the very fact for the entries t_{ij} of the transmission matrix to be i.i.d. complex gaussian variables. They consider it as the standard phenomenology and seemingly do not feel it may be worth discussing its origin. I however feel it may be deserving at least some discussion, especially as eventually the aim is to identify sources of deviations from this simple assumption. In fact such assumption corresponds to the so-called Ericsson fluctuation regime in Nuclear Physics context, see K\"{o}hness et al. https://arxiv.org/abs/2603.12068 for a recent discussion and analytic justification within microscopic Random Matrix (RM) model for chaotic scattering. Deviations of transmission statistics from Gaussian assumptions
(or closely related deviations of intensity statistics inside the scattering medium from the Rayleigh law, see Y.V. Fyodorov and E. Safonova, Phys. Rev. E 108, 044206 (2023)) are possible even without diffusion corrections if the system is nearly closed: the number of open scattering channels is of order of unity, see the above. Although such a situation appears less relevant to the current body of experiments discussed in wavefront shaping it is not impossible, and adding the corresponding discussion could help readers better appreciate the context and limitations of the background model.

Reviewer #3

(Remarks to the Author)
In this paper, the authors study wavefront shaping in complex media through theoretical, computational, and experimental efforts. Their goal is to better understand fluctuations of the enhancement factor in multi-point focusing through complex media. Their work goes beyond currently established results for the mean and fluctuations of the enhancement factor and demonstrate giant fluctuations of the factor that they attribute to long range mesoscopic correlations.

My impression is that the work is novel, interesting, and correct to the best of my knowledge. The work is well-rounded and the combination of theoretical, simulation and experiment strengthens the results and the analysis.

This work will definitely be of great significance to researchers working on wavefront shaping in complex media; it is unclear to me whether it will have interest to people working outside that area, though I would note that anyone working on light propagation in random media, whether optimizing wavefronts or not, will find the observations on mesoscopic correlations relevant.

The methodology appears sound and from what I can tell it is described sufficiently well in the Supplementary Material to be reproduced.

My impression is that this paper meets the standards for publication.

Version 1:

Reviewer comments:

Reviewer #1

(Remarks to the Author)
The authors have carefully addressed all of the referees’ comments and recommendations. The revised manuscript is

substantially clearer, better organized, and more precise in presenting both its scope and its main contribution. I am satisfied with the revisions and believe that the paper is now suitable for publication.

Reviewer #2

(Remarks to the Author)
The authors took referee's remarks very seriously and meticulously amended their presentation along all suggested lines. This considerably improved the quality of the presentation, and the paper's messages now look to me clear and convincing. I recommend publication in the present form.

Open Access This Peer Review File is licensed under a Creative Commons Attribution 4.0 International License, which permits use, sharing, adaptation, distribution and reproduction in any medium or format, as long as you give appropriate credit to the original author(s) and the source, provide a link to the Creative Commons license, and indicate if changes were made.
In cases where reviewers are anonymous, credit should be given to 'Anonymous Referee' and the source.
The images or other third party material in this Peer Review File are included in the article's Creative Commons license, unless indicated otherwise in a credit line to the material. If material is not included in the article's Creative Commons license and your intended use is not permitted by statutory regulation or exceeds the permitted use, you will need to obtain permission directly from the copyright holder.
To view a copy of this license, visit https://creativecommons.org/licenses/by/4.0/

Response to Reviewers' reports

We thank the three reviewers for their careful evaluation of our manuscript, "Largest eigenvalue statistics of wavefront shaping in complex scattering media." Their thoughtful comments and constructive suggestions have led to substantial improvements in the presentation, clarity, and overall positioning of the manuscript. We have carefully considered all of their comments and revised the manuscript accordingly.

Below, we provide a detailed point-by-point response to each comment, indicating the corresponding revisions made in the manuscript and clarifying key points where appropriate. For ease of reference, all modifications made in response to the reviewers' comments are highlighted in blue in the revised manuscript.

Reply to Reviewer 1

I read this paper with much interest and I see the potential for publication in Nature Communications. However, I feel an extensive rewriting is necessary to convey the main message in a clear and unambiguous way, and to remove parts that are quite unclear even after a few careful readings.

The main message of the paper is that experimentally accessible fluctuations of the enhancement factor already provide a sensitive probe of long-range correlations and that the enhancement factor is in fact a powerful statistical observable that encodes long-range mesoscopic correlations in complex media. This result is achieved appealing to the finite-N theory of extreme values (largest eigenvalue) for Wishart-Laguerre matrices, whose cumulative distribution can be written as a Hankel determinant thanks to the Andréief formula.

I will formulate a few comments, essentially in chronological order:

Authors' reply: We thank the referee for this thoughtful assessment and for recognizing the publication potential of our work in Nature Communications. We appreciate the suggestion that the manuscript would benefit from a clearer and more accessible presentation. Accordingly, we have substantially revised the manuscript to improve its organization, clarity, and overall presentation while more clearly emphasizing the main message. We address the referee's specific comments below.

1. Stronger effort is needed in the abstract and the introduction to set the stage and describe the relevant physics. What types of 'waves' are we talking about? What is a 'complex medium'? The narrative becomes too technical too quickly. NatComm is a more generalist journal, which should be accessible to a wide array of scientists from different disciplines. Try to make sure that each sentence leads seamlessly to the next, and that all concepts/definitions/transitions are made crystal clear for any intelligent but non-specialist reader. Writing for these journals requires adopting a different perspective - your way of writing belongs more to a 'standard' physics journal audience (like PRE, PRB, or JPhysA) but will not be as attractive for this journal's readership.

Authors' reply: In response to these comments, we have completely rewritten the Abstract and substantially revised the Introduction to provide a clearer physical motivation, introduce the key concepts more gradually, and improve the overall narrative of the manuscript. We have also revised the title to explicitly refer to complex scattering media, making the physical scope of the work more immediately apparent.

In particular, we now introduce wavefront shaping, complex scattering media, and the enhancement factor in a more accessible manner before presenting the theoretical framework. We have expanded the discussion of long-range mesoscopic correlations by placing them in a broader mesoscopic-physics context and more clearly distinguish the established theoretical framework from the conceptual advances of the present work. We also added a roadmap paragraph at the end of the Introduction to better guide readers through the organization of the manuscript.

The Abstract has been completely rewritten, and all additions and revisions made to the Introduction in response to the referee's comments are highlighted in blue in the revised manuscript.

2. Before eq. (1), you state that $\langle \lambda_{\max} \rangle$ is given by the celebrated Marčenko-Pastur law. Probably you meant that the upper edge of the support of the MP law identifies the typical location of the largest eigenvalue of large Wishart matrices.

Authors' reply: We have revised the sentence in the main text to state that, in the large M_1 and M_2 limit with fixed ratio, $\langle \lambda_{\max} \rangle$ coincides with the upper edge of the Marčenko–Pastur spectrum.

3. "We introduce a random matrix formalism" is a bit overblown. That the focusing operators constitute a Wishart ensemble is quite obvious and well-established with your assumptions - you can hardly claim that you introduced this model yourselves.

Authors' reply: We have revised the wording to more accurately reflect the scope of our contribution. Specifically, we no longer state that we introduce a new random-matrix formalism, but instead clarify that, under the standard Gaussian

assumptions adopted throughout this work, the focusing operators form a Laguerre–Wishart ensemble. We have also added Ref. [46] to acknowledge this established framework. The corresponding revisions are highlighted in blue in the main manuscript.

4. The issue of 'long-range (mesoscopic) correlations' is central to the paper, but it is introduced very hastily on p. 2. Please explain very clearly what these are, when they affect your WL assumptions and when they don't etc. Do not assume that the reader will be familiar with any of these concepts. More seriously, throughout the paper I have been very confused by two incarnations of the term 'correlations' which are often conflated, but (in my view) should instead be kept and discussed separately. One is the 'physical' level of mesoscopic correlations in your scattering problem (still to be defined and discussed better), the other is the 'mathematical'/probabilistic level, which treats the entries of your random matrix as independent (no 'correlations') and complex Gaussians. You seem to be arguing that the 'classical' WL ensemble (with independent entries) would correspond to the absence of physical mesoscopic correlations, but (if I understood correctly at all) this claim should be made way more evident, with a stronger justification. Conversely, it is not clear to me what the *presence* of mesoscopic correlations in your physical system would translate to in your random model. Would these be well described by a correlated Wishart ensemble, then? If yes, what should be the correlation matrix between Gaussian entries that correspond to a given level/pattern of physical correlations (however these are defined and measured)? These questions must absolutely be ironed out in detail.

Authors' reply: We agree that the original manuscript did not sufficiently distinguish between two different notions of correlations: (i) the long-range mesoscopic correlations arising from coherent multiple scattering in the physical system, and (ii) statistical correlations between matrix elements in a random-matrix description.

To address this point, we have substantially expanded the discussion of long-range mesoscopic correlations in both the Introduction and the Discussion. In particular, we now explain their physical origin, clarify their role in wavefront-shaping experiments, and explicitly distinguish them from the correlation-free Laguerre–Wishart (LW) ensemble, which serves as the parameter-free baseline model throughout this work.

We also added a discussion of correlated Wishart ensembles and explained why the long-range mesoscopic correlations considered here cannot, in general, be represented solely through a second-order covariance matrix. Instead, they originate from coherent multiple scattering and are characterized by connected four-field correlation functions (i.e., fourth-order cumulants), rather than by second-order covariance matrices alone. Consequently, establishing a predictive mapping between mesoscopic multiple-scattering theory and correlated random-matrix theory remains an important open problem that goes beyond the scope of the present work.

The corresponding revisions are highlighted in blue in the revised manuscript.

5. As a further general point, the narrative becomes rapidly quite messy when it comes to theory vs numerics vs experimental results. All these are often conflated inside dense paragraphs, where the different 'souls' of your paper are sometimes harder to disentangle. Details about experimental setups often appear alongside numerical simulations and theoretical derivations, making some sections hard to follow. I think presentation-wise it may be more useful to clearly have separate sub-sections (Theory - Numerical Simulations - Experimental results) or - at the very least - a 'roadmap' section where you actually guide the reader in a schematic way on what you did for each of those components.

Authors' reply: Following the referee's suggestion, we have added a roadmap paragraph at the end of the Introduction to guide readers through the organization of the manuscript and the purpose of each section. We believe that this addition makes the overall narrative significantly easier to follow. The new paragraph is highlighted in blue in the revised manuscript.

6. Fig. 2 (a) and (b). The 'clear deviation' between the Marčenko-Pastur and your LW analytical prediction is hard to assess without error bars on the experimental results. I have no problem in believing that the finite-N formulae should describe the data 'better', but your claim needs more evidence to be substantiated - after all, it may well be that, with the current experimental precision, the MP line does already a perfectly good job, which would severely weaken your claim.

Authors' reply: Following the referee's recommendation, we have added error bars to the experimental data in Figs. 2(a) and 2(b). The error bars are computed from the standard deviation of the mean enhancement factor obtained from three independent experimental transmission-matrix measurements, and therefore reflect the experimental reproducibility of the measurements rather than fluctuations between different submatrices of a single transmission matrix.

As shown in the revised figures 2(a) and 2(b), the experimental uncertainty is substantially smaller than the symbol size. Importantly, the Marčenko–Pastur prediction remains clearly outside the experimental error bars, whereas the finite-size Laguerre–Wishart prediction is in excellent agreement with the measurements. We believe that this additional analysis provides stronger experimental support for our claim that the finite-size theory is required to accurately describe the enhancement factor in finite-channel wavefront-shaping experiments. The corresponding revisions in the Fig. 2 caption are highlighted in blue in the revised manuscript.

7. I am confused again by your statement on p. 3 ‘as M1 increases, the experimental distribution broadens relative to the theoretical one’ - the way I read this sentence is: ‘there is a worrying growing discrepancy between our theory and the experiments’, which I don’t think is the message that you actually wanted to convey! What I don’t understand is: if you have a ‘better’ theory for finite M1,M2 than MP, this theory should work better across *all* values of experimentally tested M1,M2, shouldn’t it? You should not see a discrepancy between your theory and the experiments as M1 increases. I must be missing something here.

Authors’ reply: We thank the referee for pointing out this source of confusion. The Laguerre–Wishart (LW) theory does not become less accurate as M_1 increases. Rather, it provides the appropriate parameter-free baseline in the absence of long-range mesoscopic correlations.

In the experiments, however, long-range mesoscopic correlations are always present. Their influence becomes increasingly apparent as the number of controlled input channels M_1 increases, since a larger input space probes a larger fraction of the available scattering channels and therefore more effectively reveals these correlations Goetschy and Stone (2013); Hsu et al. (2017); Popoff et al. (2014); Shaughnessy et al. (2024); Vellekoop and Mosk (2008). Consequently, the experimentally measured enhancement-factor distribution becomes progressively broader than the LW prediction, not because the theory deteriorates, but because the experiments move further away from the correlation-free limit assumed by the model.

To avoid this misunderstanding, we have revised the manuscript to clarify this point at its first occurrence and explicitly state that the observed broadening reflects the increasing influence of long-range mesoscopic correlations rather than a limitation of the LW framework. The corresponding revisions are highlighted in blue in the revised manuscript.

8. For fig. 2 (c) and (d), it would be helpful to provide a quantitative measure of non-Gaussianity of the blue curves. The way the results are plotted makes it unclear whether the theoretical curves are somewhat ‘skewed’ (asymmetric tails) and by how much. I am worried that a simple Gaussian fit with appropriate mean and variance would already fit the data exceedingly well - or at least as well as your theory - without the need of a very sophisticated finite-N Random Matrix theory. If this is not the case, though, it should be made quantitatively clear here.

Authors’ reply: To quantify the non-Gaussianity of the exact finite-size distribution shown in Figs. 2(c,d), we have computed the standardized skewness and excess kurtosis from the exact finite-size distribution of $\lambda_{\max}$. Denoting

$$
\begin{aligned}
\mu &= \langle \lambda_{\max} \rangle \\
\sigma^2 &= \langle (\lambda_{\max} - \mu)^2 \rangle
\end{aligned}
$$

we define

$$
\begin{aligned}
\gamma_1 &= \frac{\langle (\lambda_{\max} - \mu)^3 \rangle}{\sigma^3} \\
\gamma_2 &= \frac{\langle (\lambda_{\max} - \mu)^4 \rangle}{\sigma^4} - 3
\end{aligned}
$$

For a Gaussian distribution, one has $\gamma_1 = \gamma_2 = 0$. The values obtained from the exact distribution are reported below, for $M_2 = 1, \ldots, 5$ and three representative values of M_1.

	$M_1 = 100$		$M_1 = 250$		$M_1 = 500$	
M_2	γ_1	γ_2	γ_1	γ_2	γ_1	γ_2
1	0.2000	0.0600	0.1265	0.0240	0.0894	0.0120
2	0.2488	0.1048	0.1900	0.0618	0.1597	0.0436
3	0.2659	0.1223	0.2141	0.0796	0.1870	0.0604
4	0.2739	0.1313	0.2266	0.0900	0.2017	0.0708
5	0.2783	0.1365	0.2343	0.0968	0.2108	0.0779

These results show that the distributions are mildly but *systematically non-Gaussian*, with positive skewness and excess kurtosis. At fixed M_2, both quantities decrease with increasing M_1, while remaining clearly nonzero for $M_2 \geq 1$. This behavior is consistent with the large M_1, fixed M_2 limit, in which the centered and rescaled largest eigenvalue converges to the largest eigenvalue of a finite-dimensional GUE matrix, whose distribution is itself non-Gaussian.

This moment-based characterization is also consistent with the large-x asymptotic behavior already derived in the previous version of the manuscript from the exact expression for $Q_{\max}(x)$. Indeed, as $x \to \infty$,

$$
1 - Q_{\max}(x) \sim x^{M_1 + M_2 - 2} e^{-M_2 x} .
$$

Thus the far-right tail is exponential, up to algebraic prefactors, rather than Gaussian. This provides an additional analytical indication of the non-Gaussian character of the distributions displayed in Figs. 2(c,d), and is fully consistent with the positive skewness and positive excess kurtosis reported above.

We would also like to clarify the role of the finite-size Laguerre–Wishart theory in the present work. Indeed, the blue curves in Figs. 2(c,d) are not empirical fits to the experimental data. Rather, they are exact, parameter-free predictions obtained from finite-size random matrix theory under the assumption that long-range mesoscopic correlations are absent.

The purpose of our theory is therefore not to provide the best phenomenological fit to the experimental distributions, but to establish a predictive baseline against which the experiments can be compared. In regimes where the influence of long-range mesoscopic correlations is negligible (e.g., for relatively small values of the number of controlled input channels M_1), the experimental distributions agree quantitatively with these predictions. In contrast, the systematic deviations observed for larger values of M_1 constitute the central finding of the present work and provide direct evidence for the influence of long-range mesoscopic correlations.

A Gaussian distribution with the same mean and variance may reproduce part of the observed shape. However, such a distribution is merely an empirical approximation, whereas the finite-size Laguerre–Wishart theory provides the exact probability distribution predicted by the underlying random-matrix model, without introducing any fitting parameters. Consequently, the purpose of the present theory is not to replace an empirical Gaussian approximation, but to provide a predictive statistical baseline against which experimentally observed correlation-induced deviations can be identified.

9. Theoretical advancements. When I started reading this paper, I was excited and expected to see a somewhat 'novel' random matrix theory of the focusing operator and the statistics of its largest eigenvalue. I was somewhat disappointed, though, because all the theoretical finite-N results are well-known from the standard theory of unitary invariant ensembles - the determinant expression in (4) is well-known and follows from a one-liner derivation using the Andréief formula on the jpdf (2) integrated between 0 and x. Moreover, the evaluation of the determinant needs anyway to be done 'numerically' (or at least with the use of symbolic software) and - as the authors correctly hint - it becomes presumably quite unwieldy for increasing M2 anyway. This is not a complete deal-breaker for the publication potential of this paper, of course, but it strongly diminishes its impact: it is somewhat disheartening that the 'waves-in-random-media' community has not bothered thus far to go beyond Marčenko and Pastur at all, and it is a good service to that community to point out that finite-N formulae exist that do a better job - but clearly this is not 'as strong as' producing a better Random Matrix theory for the focusing operator, for instance incorporating the effects of mesoscopic correlations (see point 4 above), which - in the authors' words - remains an open problem.

Authors' reply: We fully agree that the finite-size results for the Laguerre–Wishart ensemble are well established within random matrix theory and do not constitute a new mathematical development. This was already acknowledged in the original manuscript, where we cited Refs. [66] and [67] (now Refs. [80] and [81]) in this context. Our goal was therefore not to introduce a new random-matrix ensemble or derive new results within random matrix theory.

Rather, the novelty of the present work lies in demonstrating that these finite-size random-matrix results have important and previously unexplored consequences for wavefront shaping and mesoscopic optics. In particular, we show that the enhancement factor is not merely a measure of focusing performance, but also a statistical observable whose fluctuations encode long-range mesoscopic correlations. Within this framework, the finite-size Laguerre–Wishart theory provides an exact, parameter-free predictive baseline against which experimentally and numerically observed excess fluctuations can be identified.

To better emphasize this point, we have substantially revised the Abstract and the Introduction to clearly distinguish the conceptual novelty of the present work from the underlying mathematical results of random matrix theory. We now place greater emphasis on the physical significance of the enhancement factor and its fluctuations, and explicitly identify the development of a predictive correlated random-matrix theory incorporating long-range mesoscopic correlations as an important open problem beyond the scope of the present work.

10. Further on my points 4. and 9. that actually nag me quite a bit. If my intuition is correct, then a 'better' random matrix model in presence of mesoscopic correlations should be a so-called correlated Wishart ensemble, i.e. a correlation matrix of a finite number of Gaussians whose theoretical covariance is not a multiple of the identity. Does this make sense? If so, what would be a better model for such theoretical covariance? What is known about the largest eigenvalue for finite-N of correlated Wishart matrices? A quick search returned just a few hits - not much indeed, but not zero results either… your numerical experiments (at least) or - better - your theory would greatly benefit from a comparison with the correlated Wishart largest eigenvalue results (obviously, if the mapping between experimental setting and type of theoretical correlation matrix between Gaussian elements could be established or at least estimated).

Authors' reply: The referee raises an interesting question regarding the connection between long-range mesoscopic correlations and correlated Wishart ensembles. Such ensembles indeed constitute a natural mathematical generalization of the classical Laguerre–Wishart ensemble, in which statistical correlations are introduced through the second-order covariance matrix of the Gaussian transmission coefficients, e.g., $\langle t^*_{j_1 i_1} t_{i_2 j_2} \rangle$ Burda et al. (2005); Simon and Moustakas (2004). However, the long-range mesoscopic correlations considered in the present work originate from coherent multiple scattering and are characterized by higher-order connected four-field correlation functions, e.g., $\langle t^*_{j_1 i_1} t_{i_2 j_2} t^*_{j_3 i_3} t_{i_4 j_4} \rangle$ Akkermans and Montambaux (2007); Berkovits and Feng (1994); van Rossum and Nieuwenhuizen (1999), rather than solely by second-

order covariance functions. Consequently, these physical correlations cannot, in general, be represented solely through a prescribed covariance matrix for Gaussian transmission coefficients.

Establishing a predictive mapping between long-range mesoscopic correlations in multiple scattering and an effective correlated Wishart ensemble would require the development of a microscopic theory bridging mesoscopic multiple-scattering physics and correlated random-matrix theory. To the best of our knowledge, no such predictive framework is currently available. Consequently, while correlated Wishart ensembles represent an interesting direction for future investigation, developing such a framework lies well beyond the scope of the present work.

To clarify this important point, we have added a new discussion in the Discussion and Conclusions section, where we explicitly distinguish second-order covariance structures from the higher-order mesoscopic correlations investigated here and discuss the relationship between correlated Wishart ensembles and long-range mesoscopic correlations. We also identify the development of a predictive correlated random-matrix theory for wavefront-shaping experiments as an important open problem.

Finally, the objective of the present work is to establish the exact finite-size Laguerre–Wishart theory as a parameter-free predictive baseline in the absence of long-range mesoscopic correlations, and to identify experimentally and numerically the excess fluctuations induced by these correlations.

11. Another weak point of your enterprise is the fact that - while you proved that ‘long-range mesoscopic correlations’ in your physical/experimental setup can be probed by fluctuations of the enhancement factor in conjunction with your finite-M1 theory for ‘uncorrelated Gaussians’ - there doesn’t seem to be a quantitative mapping between ‘how much the discrepancy is’ in Fig. 5 (c) and (d) between the solid black line with circles and the dashed line, and the ‘amount’ of mesoscopic correlations (can this ‘amount’ be independently quantified somehow?). The argument seems to be: experiments and classical WL theory align ‘less and less’ as we tweak the experiments in such a way that we enhance these ethereal mesoscopic correlations. Ergo, this misalignment can be taken as a probe of the presence and strength of such mesoscopic correlations. But we don’t seem to have a ‘conversion table’ that tells us how much discrepancy translates into how much mesoscopic correlations - also because it is not clear to me still what these mesoscopic correlations are, and how one would measure these independently.

Authors’ reply: The objective of the present work is not to establish a quantitative conversion between the excess fluctuations beyond the Laguerre–Wishart prediction and an independently calibrated measure of the strength of long-range mesoscopic correlations. Rather, the finite-size Laguerre–Wishart theory provides a parameter-free predictive baseline for enhancement-factor statistics in the absence of long-range mesoscopic correlations. Systematic excess fluctuations beyond this baseline therefore identify the presence of additional correlations in the physical transmission matrix.

In both the experiments and the numerical simulations, these excess fluctuations become progressively larger as the number of controlled input channels M_1 increases. In the numerical simulations, the same trend is further reinforced by increasing the slab thickness, which is known to enhance long-range mesoscopic correlations while leaving the mean enhancement essentially unchanged. Together, these independent observations consistently provide the observed excess fluctuations as strong evidence of long-range mesoscopic correlations.

Establishing a quantitative mapping between enhancement-factor fluctuations and the underlying mesoscopic correlation functions would require a predictive microscopic theory relating wavefront-shaping statistics to mesoscopic multiple-scattering theory. As discussed in the revised Discussion and Conclusions section, we have explicitly addressed this point and identified the development of such a predictive framework as an important open problem beyond the scope of the present work.

In summary, I see the potential for this paper to be eventually published in NatComm, if the authors can (i) robustly revise their narrative, making the paper much more accessible and streamlined, with technical details that obstruct a seamless reading relegated to a Methods section, or an appendix at the end, and (ii) clearly stating their innovative contribution in each of the components of their work (theory - numerical simulations - experiments), bearing in mind that the theoretical treatment exhibits an obvious weakness in terms of ‘novelty’ (at least to a trained RMT eye) if not reinforced by some considerations on a correlated Wishart model (or similar).

Authors’ reply: We thank the referee for this thoughtful summary and for recognizing the potential of our work for publication in Nature Communications. Following the referee’s suggestions, we have substantially revised the manuscript to improve its accessibility, organization, and overall narrative. In particular, we have completely rewritten the Abstract, substantially revised the Introduction, clarified the physical motivation and key concepts, revised the title to better reflect the scope of the work, and added a roadmap paragraph to guide readers through the organization of the manuscript. We have also strengthened the Discussion and Conclusions section by clarifying the physical interpretation of long-range mesoscopic correlations, discussing the relation to correlated Wishart ensembles, and identifying the development of a predictive correlated random-matrix theory as an important open problem.

We have also substantially revised the presentation of the work to more clearly distinguish its conceptual novelty from the underlying mathematical results of random matrix theory. Throughout the revised manuscript, we now place greater

emphasis on the physical significance of the enhancement factor and its fluctuations, and explicitly identify the principal contribution of this work as establishing enhancement-factor fluctuations as a simple and experimentally accessible probe of long-range mesoscopic correlations.

We hope that these revisions have addressed the referee's concerns and significantly strengthened the manuscript.

Typo: confront —¿ compare (p. 5)

Authors' reply: Corrected as suggested.

Reply to Reviewer 2

The paper develops a comprehensive analysis of the fluctuations of the enhancement factor in wave focussing experiments in a complex diffusive medium. Theoretical part is based on assuming the entries t_{ij} of the associated transmission matrix to be i.i.d. complex gaussian variables. Such an assumption is well-justified in very open (many channel) medium in the regime of fully ergodic chaotic scattering. However in the diffusive regime it may be violated via the influence of mesoscopic long-range correlations in transmission amplitudes. The authors' approach is simple but powerful: using the above assumptions for building a suitable random matrix zero-level model, they aim at characterizing the statistics of the largest eigenvalue of the focussing operator built from $M_2 \times M_1$ submatrices of the transmission matrix, with M_2 being the number of focussing targets (generally, of the order of unity), while $M_1 \gg M_2$ is the number of controlled input channels. The corresponding focussing operator is then represented by the so-called anti-Wishart random matrix of unitary symmetry class whose eigenvalues for any size are described by a determinantal process with explicitly known kernel. Employing the standard random matrix theory (RMT) allows to represent the cumulative probability function for the largest eigenvalue as a finite-size determinant, providing a tool for its efficient evaluation. This is a relatively rare example when RMT proves to be useful beyond the large size asymptotic analysis. Having such exact representation is crucial for the ultimate goal of the paper: comparison with experimental data on focussing. Such a comparison reveals that with increase in scattering medium widths the predictions of developed theory start showing more and more considerable deviations from the experimental data. The authors very reasonably attribute such discrepancy to the influence of mentioned long-range mesoscopic correlations neglected in the underlying assumptions on transmission matrix statistics. In this way their approach offers a sensitive test for such correlations, which otherwise are quite difficult to measure. Although none of the theoretical ingredients employed to arrive to the conclusion is particularly new, the very idea and its skillful implementation in the paper, both theoretical and experimental, look to me certainly worthy of publications in Nature Communication.

Authors' reply: We thank the referee for this thoughtful and positive assessment of our work. We are pleased that the referee appreciated both the theoretical framework and the experimental validation presented in the manuscript. In particular, we are encouraged that the referee recognized the value of combining finite-size random matrix theory with wavefront-shaping experiments to characterize enhancement-factor fluctuations and probe long-range mesoscopic correlations in complex media. We also appreciate the referee's assessment that the significance of the work lies not in the novelty of the individual theoretical ingredients, but rather in the underlying idea and its implementation through the combination of theory and experiment.

A minor remark is due.

The authors take for granted the very fact for the entries t_{ij} of the transmission matrix to be i.i.d. complex gaussian variables. They consider it as the standard phenomenology and seemingly do not feel it may be worth discussing its origin. I however feel it may be deserving at least some discussion, especially as eventually the aim is to identify sources of deviations from this simple assumption. In fact such assumption corresponds to the so-called Ericsson fluctuation regime in Nuclear Physics context, see Köhness et al. https://arxiv.org/abs/2603.12068 for a recent discussion and analytic justification within microscopic Random Matrix (RM) model for chaotic scattering. Deviations of transmission statistics from Gaussian assumptions (or closely related deviations of intensity statistics inside the scattering medium from the Rayleigh law, see Y.V. Fyodorov and E. Safonova, Phys. Rev. E 108, 044206 (2023)) are possible even without diffusion corrections if the system is nearly closed: the number of open scattering channels is of order of unity, see the above. Although such a situation appears less relevant to the current body of experiments discussed in wavefront shaping it is not impossible, and adding the corresponding discussion could help readers better appreciate the context and limitations of the background model.

Authors' reply: Following the referee's suggestion, we have expanded the discussion of the physical motivation underlying the assumption of i.i.d. complex Gaussian transmission coefficients. In particular, we now explain that Gaussian statistics of transmission amplitudes provide the standard baseline description of highly open scattering systems supporting a large number of channels. We further discuss how the coherent superposition of many scattering paths gives rise to fully developed speckle and approximately Gaussian field statistics, thereby motivating the Laguerre–Wishart framework adopted throughout this work. We have also added the references suggested by the referee, together with additional review articles that provide broader context on random-matrix approaches to open scattering systems.

The corresponding discussion has been added at the beginning of the *Random-Matrix Framework: The Laguerre–Wishart Ensemble* section and is highlighted in blue in the revised manuscript.

Reply to Reviewer 3

In this paper, the authors study wavefront shaping in complex media through theoretical, computational, and experimental efforts. Their goal is to better understand fluctuations of the enhancement factor in multi-point focusing through complex media. Their work goes beyond currently established results for the mean and fluctuations of the enhancement factor and demonstrate giant fluctuations of the factor that they attribute to long range mesoscopic correlations.

My impression is that the work is novel, interesting, and correct to the best of my knowledge. The work is well-rounded and the combination of theoretical, simulation and experiment strengthens the results and the analysis.

Authors' reply: We thank the referee for this careful and encouraging assessment of our work. We are pleased that the referee found the study to be novel, interesting, and technically sound. In particular, we appreciate the referee's recognition that the manuscript goes beyond the currently established understanding of enhancement-factor statistics and reveals giant fluctuations associated with long-range mesoscopic correlations.

We are also encouraged that the referee viewed the combination of theory, numerical simulations, and experiments as a strength of the work and as an important element supporting the validity of our conclusions.

This work will definitely be of great significance to researchers working on wavefront shaping in complex media; it is unclear to me whether it will have interest to people working outside that area, though I would note that anyone working on light propagation in random media, whether optimizing wavefronts or not, will find the observations on mesoscopic correlations relevant.

The methodology appears sound and from what I can tell it is described sufficiently well in the Supplementary Material to be reproduced.

My impression is that this paper meets the standards for publication.

Authors' reply: We thank the referee for these encouraging remarks. We are pleased that the referee recognizes the potential significance of our findings for the wavefront-shaping community, as well as the broader relevance of mesoscopic correlations to the study of wave propagation in complex media. We also appreciate the referee's assessment that the methodology is sound and described in sufficient detail to enable reproducibility.

While the present work is motivated by wavefront-shaping applications, we agree that the underlying physics of long-range mesoscopic correlations extends beyond this specific context and is relevant to a broader range of problems involving wave transport in disordered media.

References

Akkermans, E. and Montambaux, G. (2007). *Mesoscopic Physics of Electrons and Photons*. Cambridge Univ. Press.

Berkovits, R. and Feng, S. (1994). Correlations in coherent multiple scattering. *Phys. Rep.*, 238(3):135–172.

Burda, Z., Jurkiewicz, J., and Wacław, B. (2005). Spectral moments of correlated Wishart matrices. *Phys. Rev. E*, 71(2):026111.

Goetschy, A. and Stone, A. D. (2013). Filtering random matrices: the effect of incomplete channel control in multiple scattering. *Phys. Rev. Lett.*, 111:063901.

Hsu, C. W., Liew, S. F., Goetschy, A., Cao, H., and Stone, A. D. (2017). Correlation-enhanced control of wave focusing in disordered media. *Nat. Phys.*, 13:497–502.

Popoff, S. M., Goetschy, A., Liew, S. F., Stone, A. D., and Cao, H. (2014). Coherent control of total transmission of light through disordered media. *Phys. Rev. Lett.*, 112:133903.

Shaughnessy, L., McIntosh, R. E., Goetschy, A., Hsu, C. W., Bender, N., Yılmaz, H., Yamilov, A., and Cao, H. (2024). Multiregion light control in diffusive media via wavefront shaping. *Phys. Rev. Lett.*, 133(14):146901.

Simon, S. H. and Moustakas, A. L. (2004). Eigenvalue density of correlated complex random Wishart matrices. *Phys. Rev. E*, 69(6):065101.

van Rossum, M. C. W. and Nieuwenhuizen, T. M. (1999). Multiple scattering of classical waves: microscopy, mesoscopy, and diffusion. *Rev. Mod. Phys.*, 71(1):313.

Vellekoop, I. M. and Mosk, A. P. (2008). Universal optimal transmission of light through disordered materials. *Phys. Rev. Lett.*, 101:120601.
